# ﻿Annotation and functional prediction of RNA helicases in *Ustilago maydis*

**DOI:** 10.3897/imafungus.16.151785

**Published:** 2025-10-02

**Authors:** Amanda M. Seto, Barry J. Saville

**Affiliations:** 1 Environmental and Life Sciences Graduate Program, Trent University, Peterborough, ON, K9L 0G2, Canada Trent University Peterborough Canada; 2 Department of Forensic Science, Trent University, Peterborough, ON, K9L 0G2, Canada Trent University Peterborough Canada

**Keywords:** Control of pathogenic development, genome annotation, RNA helicases, smut fungi, *Ustilago maydis*

## Abstract

RNA helicases are conserved enzymes found in both prokaryotes and eukaryotes. They function in all aspects of RNA metabolism and are known to influence various cellular and metabolic processes. In addition, they have been implicated in certain cancers and diseases. Studies on RNA helicases in fungi indicate their conserved roles in RNA metabolism and suggest that their dysregulation can affect fungal growth. However, the roles of RNA helicases in fungal plant pathogenesis remain underexplored, despite increasing knowledge of how RNA helicases modulate gene expression and disease progression. We used the basidiomycete plant pathogen *Ustilago maydis* as a model to identify 46 RNA helicases. We review the roles of RNA helicases in RNA metabolism, cellular growth and homeostasis, and metabolism. We then utilized available *U. maydis* transcriptome data and current research to hypothesize potential functions of RNA helicases in fungal plant pathology. These roles include influencing cell growth, modulating stress response, contributing to virulence and disease progression, and regulating fungal spore dormancy and germination. Understanding the roles of RNA helicases in gene regulation may aid in developing strategies to mitigate disease spread in fungal plant pathogens.

## ﻿Introduction

Helicases are conserved enzymes that utilize ATP to bind and remodel nucleic acids. These proteins are found in both prokaryotes and eukaryotes, and many exhibit conserved functions across species. DNA helicases participate in several cellular processes, including DNA replication and repair. RNA helicases are involved in all aspects of RNA metabolism, such as transcription, splicing, ribosome biogenesis, and translation (Singleton et al. 2007). A considerable amount of research supports the roles of RNA helicases in cellular metabolism, growth, and viability. An increased understanding of how defective helicases affect cellular processes has also led to the identification of their roles in certain cancers and diseases (Fairman-Williams et al. 2010; Cai et al. 2017). In fungi, functional analysis of RNA helicases has been extensively conducted in the budding yeast *Saccharomyces cerevisiae*. Investigating their roles in yeast RNA metabolism has led to the identification of RNA helicases involved in cellular growth, metabolism, and stress response. Although studies on pathogenic fungi are limited, they have revealed roles for RNA helicases in virulence (Panepinto et al. 2005; Bleichert and Baserga 2007; Delaney et al. 2013; Bohnsack et al. 2023). In all of these roles, the helicases act to unwind double-stranded RNA (dsRNA).

The separation of RNA–RNA and RNA–DNA duplexes by RNA helicases may also involve displacing proteins from RNA, acting as RNA clamps, or annealing RNA strands. Some RNA helicases are essential, and defects in these proteins can affect cell viability (Bleichert and Baserga 2007; Jankowsky 2011; Linder and Jankowsky 2011), indicating potential roles in cellular growth and homeostasis. In fungi, certain RNA helicases have been shown to modulate the expression of subsets of genes, which may influence stress responses or virulence. For example, in *Cryptococcus neoformans*, the RNA helicase Vad1 regulates several virulence-associated genes (Panepinto et al. 2005). Characterization of *ski2* deletion mutants in *C. neoformans* revealed decreased virulence, increased resistance to azoles, and sensitivity to high temperatures and osmotic stress (Li et al. 2022). In *Neurospora crassa*, the RNA helicase FRH forms a complex with FRQ and CK1a to regulate the circadian clock (Cheng et al. 2005; Lauinger et al. 2014). In *S. cerevisiae*, the nutrient stress response is modulated by the RNA helicase Dbp2 (Beck et al. 2014), which interacts with *SKS1*, a gene encoding a protein responsible for adaptation to low-glucose conditions (Paul et al. 2025). Despite current knowledge of RNA helicases in fungi, their functions in phytopathogenic fungi remain largely unexplored. Their roles in fungal growth, virulence, pathogenesis, and spore dormancy have not been extensively studied, and experimental investigations in smuts and rusts of the *Basidiomycota* are limited.

In this review, we utilize the smut fungus *Ustilago maydis* to identify potential RNA helicases of interest and hypothesize their roles in the fungal life cycle. *U. maydis* is a member of the *Basidiomycota* and belongs to the *Ustilaginales*, which includes more than 1,500 species. These species are important plant pathogens that infect members of the plant family *Poaceae*, which includes many cereal crops. *Ustilago maydis* infects the aerial parts of maize, causing common smut. These infections result in significant losses in food production, costing millions of dollars annually (Martínez-Espinoza et al. 2002; Brefort et al. 2009). Although its agricultural impact is not as substantial as that of rust fungi, *U. maydis* is considered one of the top ten fungal pathogens of scientific importance and serves as a model organism for studying plant–pathogen interactions (Dean et al. 2012). This review highlights the functions of RNA helicases in RNA metabolism, identifies RNA helicases in *U. maydis*, and discusses those that may affect fungal growth, metabolism, stress response, pathogenesis, and teliospore dormancy and germination.

## ﻿Characteristics of SF1 and SF2 RNA helicase superfamilies

**Figure 1.**
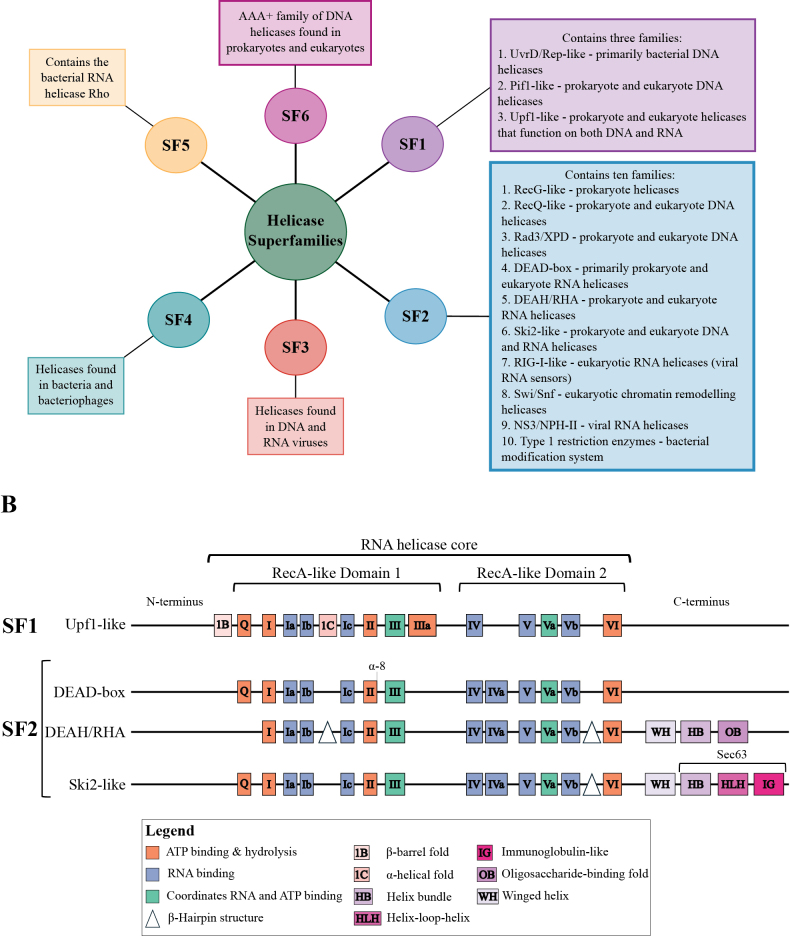
Helicase superfamilies and characteristics of SF1 and SF2 RNA helicases. **A** Summary of the six helicase superfamilies found in prokaryotes and eukaryotes; **B** The characteristics of the N-terminus, C-terminus domains, and RNA helicase core of SF1 and SF2 RNA helicases. The SF1 and SF2 RNA helicases have two RecA-like domains containing specific sequence motifs. These sequence motifs are colored according to their biochemical functions: orange, ATP binding and hydrolysis; blue, RNA binding; and green, coordination between RNA and ATP binding. The N-terminus and C-terminus of RNA helicases contain some conserved domains that are indicated; however, variability in these regions provides specificity to the RNA helicase. The α-8 represents the conserved α-helix at the end of motif II. The triangle represents the position of the β-hairpin structure motif. Abbreviations: HB, helix bundle containing the ratchet helix; HLH, helix-loop-helix; IG, immunoglobulin-like; OB, oligosaccharide-binding fold; WH, winged helix. The distance between domains and sequence motif sizes is not to scale.

Currently, six helicase superfamilies containing both DNA and RNA helicases have been identified (Fig. 1A). The largest among these are superfamilies 1 (SF1) and 2 (SF2), with the majority of eukaryotic RNA helicases classified under SF2. The remaining superfamilies (SF3–SF6) typically include enzymes involved in DNA metabolism (Singleton et al. 2007; Fairman-Williams et al. 2010). Superfamily 3 (SF3) helicases are found in DNA and RNA viruses (Hickman and Dyda 2005), while superfamily 4 (SF4) helicases occur in bacteria and bacteriophages (O’Donnell and Li 2018). Superfamily 5 (SF5) includes the bacterial RNA helicase Rho, which is responsible for transcription termination (Selvaratnam et al. 2025). Superfamily 6 (SF6), also known as the AAA+ superfamily, comprises helicases involved in DNA metabolism (Ilves et al. 2010). The classification of helicases was first proposed based on sequence and structural motifs by Gorbalenya and Koonin (1993) and was later refined through further structural and functional analyses (Singleton et al. 2007). These sequence motifs make up the helicase core, which contains two RecA-like domains. The functions of these motifs include ATP binding and hydrolysis, RNA binding, interdomain interactions, and coordination between ATP and RNA binding (Bleichert and Baserga 2007; Fairman-Williams et al. 2010). In RNA helicases, the helicase core is characterized by approximately 14 sequence motifs, as illustrated in Fig. 1B. Some motifs (e.g., I, II, and VI) are conserved across all RNA helicases, whereas others are present only in specific subsets (Fairman-Williams et al. 2010; Sloan and Bohnsack 2018). This review focuses on fungal RNA helicases belonging to the SF1 and SF2 superfamilies.

Sequence and structural analyses conducted by Gorbalenya and Koonin (1993) and Singleton et al. (2007) identified three families of helicases within SF1. These families are the UvrD/Rep-like, Pif1-like, and Upf1-like helicases. The UvrD/Rep-like family consists primarily of DNA helicases found in bacteria and a few eukaryotes (Gilhooly et al. 2013). Pif1-like helicases are found in both eukaryotes and prokaryotes. This family includes DNA helicases that promote nuclear and mitochondrial genome stability and DNA repair (Bochman et al. 2010; Gilhooly et al. 2013). SF1 RNA helicases belong to the Upf1-like family. They are conserved RNA helicases that function in nonsense-mediated RNA decay and prevent genome instability during transcription (Mischo et al. 2011; Gupta and Li 2018). These RNA helicases contain sequence domains that are conserved among the Upf1-like RNA helicases and are not found in other RNA helicase families (Fig. 1B). Subdomains 1B and 1C are located in conserved positions within the RNA helicase core (Fairman-Williams et al. 2010). The 1B subdomain forms a β-barrel fold, and 1C forms an α-helical fold within the helicase core, both contributing to modulating conformational changes in the RNA helicase (Gowravaram et al. 2018; Kanaan et al. 2018). Motif IIIa is specific to SF1 RNA helicases and serves as a stacking platform for adenine in ATP through a conserved tyrosine residue (Fairman-Williams et al. 2010).

The SF2 superfamily of RNA helicases is the largest and contains ten families of helicases (Fig. 1A). These families were identified by Fairman-Williams et al. (2010) through sequence and phylogenetic analyses. SF2 family members include RecG-like, RecQ-like, Rad3/XPD, DEAD-box, DEAH/RHA, Ski2-like, RIG-I-like, Swi/Snf, NS3/NPH-II, and Type I restriction enzymes. RecG-like helicases are found in prokaryotes, RecQ-like and Rad3/XPD families contain DNA helicases, NS3/NPH-II helicases are viral RNA helicases, and Type I restriction enzymes are part of the restriction–modification system in bacteria. Fungal SF2 RNA helicases are found among the remaining families (Fairman-Williams et al. 2010; Byrd and Raney 2012).

The DEAD-box family is the largest family of helicases, with members conserved across prokaryotes and eukaryotes. DEAD-box RNA helicases contain a helicase core composed of 12 sequence motifs. The defining feature of this family is motif II, which contains the amino acid sequence Asp-Glu-Ala-Asp (DEAD) (Fairman-Williams et al. 2010; Jankowsky 2011; Linder and Jankowsky 2011; Byrd and Raney 2012). Many DEAD-box helicases bind to RNA in an ATP-dependent manner. They modulate structured RNAs by disrupting secondary and tertiary structures and can also disrupt RNA–protein interactions in ribonucleoprotein complexes (RNPs). DEAD-box helicases can separate RNA duplexes by disrupting the duplex, thereby accelerating strand separation. This occurs in an ATP-dependent manner but is typically limited to short duplexes (Yang et al. 2007). Structurally, DEAD-box RNA helicases contain an α-helix, termed α-helix 8, located at the end of motif II (Fig. 1B). This structure is proposed to regulate access to the binding site when the helicase is in the ADP-bound state. Upon ATP binding, a conformational change occurs that moves α-helix 8 from the binding site to interact with a conserved arginine in motif V (Schütz et al. 2010; Cordin et al. 2012).

The DEAH/RNA helicase A (RHA) family contains spliceosomal and RHA-group subfamilies. Spliceosomal RNA helicases function in pre-mRNA splicing, while RHA-group helicases are involved in transcription, RNA export, and translation. The conserved sequence motifs in DEAH/RHA helicases differ from those in DEAD-box helicases, and DEAD-box helicases have a more variable C-terminal domain (Fig. 1B). In contrast, the C-terminal domain of DEAH/RHA helicases is conserved and includes a winged helix, helix bundle, and oligosaccharide-binding fold. This domain organization contributes to RNA binding strength and the helicase’s translocating activity (De Bortoli et al. 2021). The Q-motif, which is involved in ATP binding, is absent in DEAH/RHA helicases (Fairman-Williams et al. 2010). Structurally, DEAH/RHA helicases contain two β-hairpins (Fig. 1B): one in RecA-like domain 1, between motifs Ib and Ic, and another in RecA-like domain 2, between motifs Vb and VI (Cordin and Beggs 2013; He et al. 2017). The first β-hairpin is an extension of motif Ib, is unique and conserved in DEAH/RHA helicases, and is responsible for unwinding RNA in a 3′-to-5′ direction (He et al. 2017). The β-hairpin in RecA-like domain 2 is longer than that found in the same position in Ski2-like helicases. Structural analyses show that when the helicase is in the ADP state, this β-hairpin blocks access to the nucleic acid binding cavity, which is formed by the two RecA-like domains, the helix bundle, and the winged helix. ATP binding induces a conformational change that removes the β-hairpin from the cavity, allowing RecA-like domain 2 to bind to the 5′ end of the RNA duplex. In the proposed model for strand separation, the β-hairpin slices through the RNA duplex, while the helix bundle pulls the single-stranded RNA through the cavity. The OB-fold is located at the cavity entrance and serves as a docking platform for other binding proteins (He et al. 2010; Walbott et al. 2010; He et al. 2017).

The Ski2-like family of SF2 RNA helicases comprises two subfamilies: Ski2 and Brr2. This is a small family with a structure distinct from other SF2 RNA helicases. Like DEAH/RHA helicases, Ski2-like helicases possess a short β-hairpin between motifs Va and VI (Fig. 1B). This β-hairpin is proposed to function similarly to its DEAH/RHA counterpart, assisting in duplex unwinding by positioning itself between the strands (reviewed in Fairman-Williams et al. 2010; Johnson and Jackson 2013). At the C-terminus, Ski2-like helicases contain a conserved winged helix and a Sec63 domain (Fig. 1B). The Sec63 domain includes a helix bundle (HB), helix-loop-helix (HLH), and immunoglobulin-like (IG) domains. This domain is unique to the Ski2-like family and may contribute to regulating substrate binding by forming a tunnel through which RNA binds and is translocated during unwinding (Cordin and Beggs 2013).

## ﻿Identification of *Ustilago maydis* RNA helicases

RNA helicases in *Ustilago maydis* and their putative functions have not been previously investigated. Our interest in the function of *U. maydis* RNA helicases stemmed from earlier research in our laboratory, which suggested that some gene transcripts are stabilized in the dormant teliospore through the formation of double-stranded RNAs (dsRNAs). During teliospore germination, these stabilized transcripts would need to be unwound and made available for translation (Donaldson and Saville 2013; Ostrowski and Saville 2017). It was proposed that RNA helicases may modulate the availability of mRNAs by unwinding the dsRNAs in the teliospore following the initiation of germination. We identified putative *U. maydis* RNA helicases and proposed functions based on homology to previously characterized RNA helicases in other model organisms. We then selected RNA helicases with proposed roles in fungal development and growth, inferred from current research in other eukaryotes.

**Figure 2.**
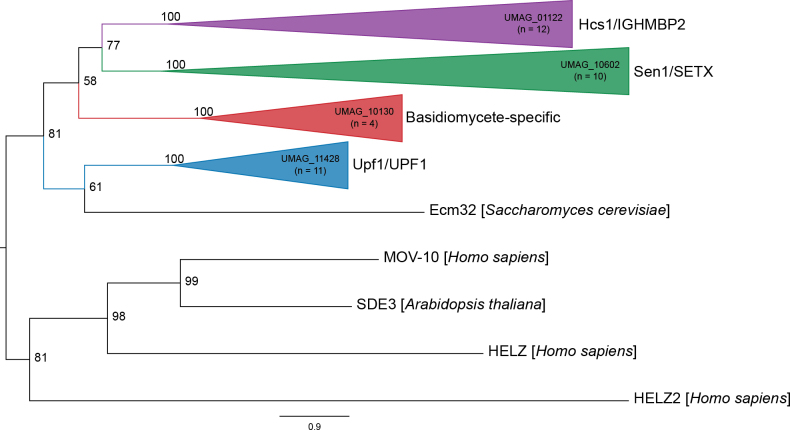
Maximum likelihood phylogenetic tree of SF1 RNA helicases. The phylogenetic tree was created with orthologs from *U. maydis*, *S. reilianum*, *U. hordei*, *C. neoformans*, *S. cerevisiae*, *S. pombe*, *N. crassa*, *A. thaliana*, *C. elegans*, *D. discoideum*, and *H. sapiens* using W-IQ-TREE multicore version 1.6.12 with default settings (Trifinopoulos et al. 2016), 1,000 ultrafast bootstrap alignments, and the approximate Bayes test. The tree was visualized with FigTree v1.4.4 (http://tree.bio.ed.ac.uk/software/figtree/), rooted at the midpoint, and bootstrap values are indicated for each node. Phylogenetic groups containing an RNA helicase ortholog in *U. maydis* were collapsed and color-coded. The *U. maydis* RNA helicase and the number of sequences for each clade are identified for each collapsed group. Each collapsed group was named after the *S. cerevisiae* and *H. sapiens* orthologs. The scale bar indicates the expected number of substitutions per amino acid. The original phylogenetic tree is in Suppl. material 1: fig. S1 and contains all gene names and organisms used in this analysis.

**Figure 3.**
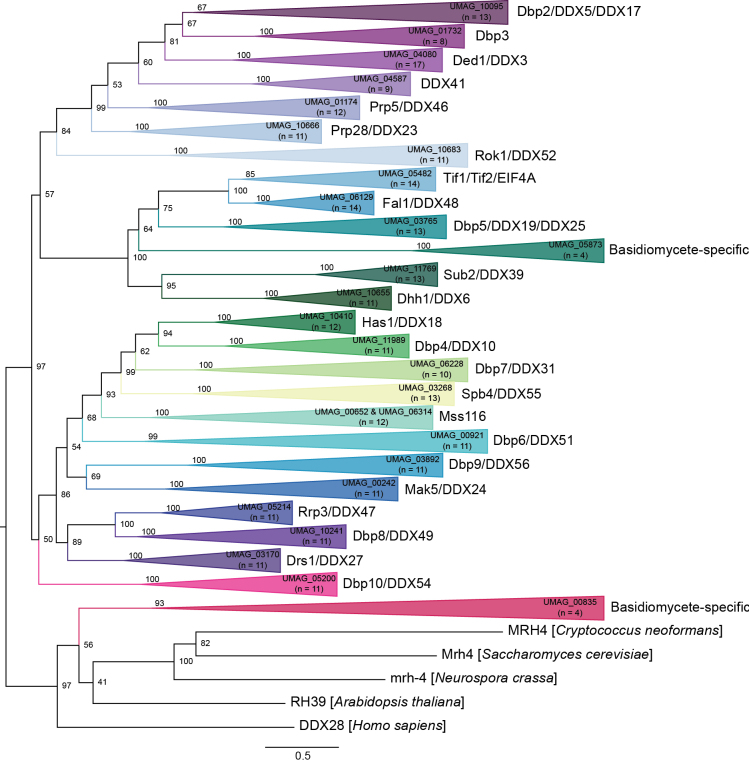
Maximum likelihood phylogenetic tree of SF2DEAD-box RNA helicases. The phylogenetic tree was created with orthologs from *U. maydis*, *S. reilianum*, *U. hordei*, *C. neoformans*, *S. cerevisiae*, *S. pombe*, *N. crassa*, *A. thaliana*, *C. elegans*, *D. discoideum*, and *H. sapiens* using W-IQ-TREE multicore version 1.6.12 with default settings (Trifinopoulos et al. 2016), 1,000 ultrafast bootstrap alignments, and the approximate Bayes test. The tree was visualized with FigTree v1.4.4 (http://tree.bio.ed.ac.uk/software/figtree/), rooted at the midpoint, and the bootstrap value is indicated for each node. Phylogenetic groups containing an RNA helicase ortholog in *U. maydis* were collapsed and color-coded. The *U. maydis* RNA helicase and the number of sequences for each clade are identified for each collapsed group. Each collapsed group was named after the *S. cerevisiae* and *H. sapiens* orthologs. The scale bar indicates the expected number of substitutions per amino acid. The original phylogenetic tree is provided in Suppl. material 1: fig. S2 and contains all gene names and organisms used in this analysis.

**Figure 4.**
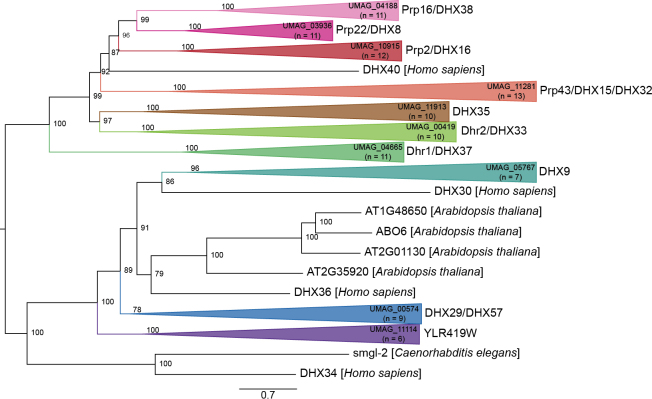
Maximum likelihood phylogenetic tree of SF2 DEAH/RHA RNA helicases. The phylogenetic tree was created with orthologs from *U. maydis*, *S. reilianum*, *U. hordei*, *C. neoformans*, *S. cerevisiae*, *S. pombe*, *N. crassa*, *A. thaliana*, *C. elegans*, *D. discoideum*, and *H. sapiens* using W-IQ-TREE multicore version 1.6.12 with default settings (Trifinopoulos et al. 2016), 1,000 ultrafast bootstrap alignments, and the approximate Bayes test. The tree was visualized with FigTree v1.4.4 (http://tree.bio.ed.ac.uk/software/figtree/), rooted at the midpoint, and the bootstrap value is indicated for each node. Phylogenetic groups containing an RNA helicase ortholog in *U. maydis* were collapsed and color-coded. The *U. maydis* RNA helicase and the number of sequences for each clade are identified for each collapsed group. Each collapsed group was named after the *S. cerevisiae* and *H. sapiens* orthologs. The scale bar indicates the expected number of substitutions per amino acid. The original phylogenetic tree is provided in Suppl. material 1: fig. S3 and contains all gene names and organisms used in this analysis.

**Figure 5.**
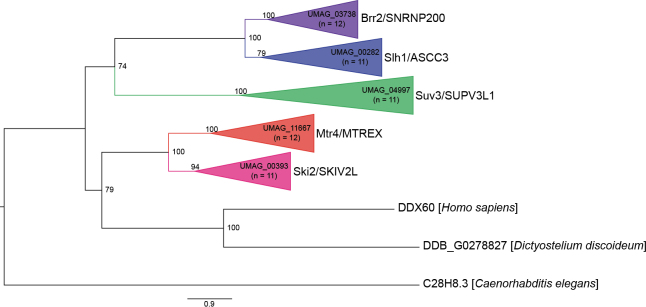
Maximum likelihood phylogenetic tree of SF2 Ski2-like RNA helicases. The phylogenetic tree was created with orthologs from *U. maydis*, *S. reilianum*, *U. hordei*, *C. neoformans*, *S. cerevisiae*, *S. pombe*, *N. crassa*, *A. thaliana*, *C. elegans*, *D. discoideum*, and *H. sapiens* using W-IQ-TREE multicore version 1.6.12 with default settings (Trifinopoulos et al. 2016), 1,000 ultrafast bootstrap alignments, and the approximate Bayes test. The tree was visualized with FigTree v1.4.4 (http://tree.bio.ed.ac.uk/software/figtree/), rooted at the midpoint, and the bootstrap value is indicated for each node. Phylogenetic groups containing an RNA helicase ortholog in *U. maydis* were collapsed and color-coded. The *U. maydis* RNA helicase and the number of sequences for each clade are identified for each collapsed group. Each collapsed group was named after the *S. cerevisiae* and *H. sapiens* orthologs. The scale bar indicates the expected number of substitutions per amino acid. The original phylogenetic tree is in Suppl. material 1: fig. S4 and contains all gene names and organisms used in this analysis.

Putative *U. maydis* RNA helicases were identified using previously characterized RNA helicases from *Saccharomyces cerevisiae*, *Caenorhabditis elegans*, *Drosophila melanogaster*, *Mus musculus*, and *Homo sapiens* (Jankowsky 2011; Bourgeois et al. 2016). Known protein sequences of these RNA helicases were compared to those of *U. maydis* using reciprocal BLASTp, which identified 46 RNA helicases in *U. maydis* (Table 1). In comparison, approximately 41 RNA helicases have been identified in *S. cerevisiae* and 70 in *H. sapiens* (Bohnsack et al. 2023). Plant species such as *Arabidopsis thaliana*, *Zea mays*, and *Oryza sativa* contain more than 150 RNA helicases (Li et al. 2023). The 46 *U. maydis* RNA helicases were categorized into the SF1 and SF2 superfamilies: four belong to the SF1 Upf1-like group, and 42 are classified within the SF2 superfamily. The SF2 helicases were further divided among the DEAD-box, DEAH/RHA, and Ski2-like families. No *U. maydis* RNA helicases were identified in the RIG-I-like family, which is expected, as RIG-I-like helicases typically act as viral sensors involved in the antiviral immune response (Byrd and Raney 2012). Additionally, although DICER1 in eukaryotes is classified as a RIG-I-like helicase due to the presence of a helicase domain (Table 1), an ortholog in *U. maydis* was not expected, as this species lacks the RNA interference (RNAi) machinery (Nakayashiki et al. 2006). Further classification of *U. maydis* RNA helicases was carried out through phylogenetic analyses using protein sequences from *U. maydis*, *Sporisorium reilianum*, *Ustilago hordei*, *S. cerevisiae*, *Schizosaccharomyces pombe*, *Neurospora crassa*, *C. elegans*, *H. sapiens*, *Dictyostelium discoideum*, and *A. thaliana*. Protein sequences were aligned using Jalview v2.11 (Waterhouse et al. 2009) and the JABAWS service to perform MUSCLE alignment under default settings (Edgar 2004; Troshin et al. 2011). Maximum likelihood phylogenetic trees were then constructed for each RNA helicase family (Figs 2–5).

**Table 1.** RNA helicases in *U. maydis* and their orthologs in *S. cerevisiae*, *C. elegans*, *D. melanogaster*, *M. musculus*, and *H. sapiens*.

Superfamily	Family	*U. maydis*	*S. cerevisiae*	*C. elegans*	*D. melanogaster*	*M. musculus*	*H. sapiens*
SF1	Upf1-like	UMAG_10602	Sen1	eri-7	CG7504	Setx	SETX
		UMAG_11428	Upf1	smg-2	Upf1	Upf1	UPF1
		UMAG_01122	Hcs1	Y106G6D.5		IGHMBP2	IGHMBP2
		UMAG_10130			CG6701	MOV10	MOV10
			Ecm32		armi	Mov10l1	MOV10L1
				C44H9.4	Helz	Helz	HELZ
						Helz2	HELZ2
				emb-4	CG14729	Aqr	AQR
SF2	DEAD-box	UMAG_05482	Tif1	inf-1	eIF4A	Ddx2a	DDX2A
			Tif2	F57B9.3		Ddx2b	DDX2B
		UMAG_06129	Fal1	F33D11.10 Y65B4A.6	eIF4A-III	DDX48	DDX48
		UMAG_10655	Dhh1	cgh-1	me3B	DDX6	DDX6
				mel-46	Gem3	DDX20	DDX20
		UMAG_03765	Dbp5	ddx-19	DBP80	DDX19a	DDX19A
						DDX19b	DDX19B
		UMAG_11769	Sub2	hel-1	Hel25E	DDX39A	DDX39A
						DDX39B	DDX39B
		UMAG_05873					
		UMAG_10410	Has1	B0511.6	CG6375	DDX18	DDX18
		UMAG_11989	Dbp4	ddx-10	CG5800	DDX10	DDX10
				Y55F3BR.1	CG9054	DDX1	DDX1
		UMAG_03268	Spb4	ZK512.2	DDX55	DDX55	DDX55
		UMAG_06228	Dbp7		CG8611	DDX31	DDX31
		UMAG_05214	Rrp3	T26G10.1	CG9253	DDX47	DDX47
		UMAG_10241	Dbp8	H20J04.4	DBP45A	DDX49	DDX49
		UMAG_03170	Drs1	ddx-27	Rs1	DDX27	DDX27
		UMAG_05200	Dbp10	Y94H6A.5	CG32344	DDX54	DDX54
		UMAG_04080	Ded1	laf-1	Belle	DDX3X	DDX3X
			Dbp1	vbh-1		DDX3Y	DDX3Y
						D1Pas1	
				glh-1	Vasa	DDX4	DDX4
				glh-2			
		UMAG_10095	Dbp2	ddx-17	Rm62	DDX5	DDX5
						DDX17	DDX17
					CG7878		DDX53
						DDX43	DDX43
		UMAG_01732	Dbp3				
		UMAG_04587		sacy-1	abs	DDX41	DDX41
		UMAG_10666	Prp28	ddx-23	CG10333	DDX23	DDX23
				C46F11.4	DmRH27	DDX42	DDX42
		UMAG_01174	Prp5	ddx-46	CG6227	DDX46	DDX46
		UMAG_10683	Rok1	ddx-52	DmRH17	DDX52	DDX52
		UMAG_03892	Dbp9	C24H12.4	CG1666	DDX56	DDX56
		UMAG_00921	Dbp6	ZK686.2	Dbp73D	DDX51	DDX51
						Ddx21	DDX21
SF2	DEAD-box					Ddx50	DDX50
		UMAG_00242	Mak5	F55F8.2			DDX24
						DDX59	DDX59
		UMAG_00652	Mss116				
		UMAG_06314					
			Mrh4		Dbp21E2	DDX28	DDX28
		UMAG_00835					
	DEAH/RHA	UMAG_10915	Prp2	mog-4	CG10689	DHX16	DHX16
		UMAG_04188	Prp16	mog-1	CG32604	DHX38	DHX38
		UMAG_03936	Prp22	mog-5	CG8241	DHX8	DHX8
		UMAG_11281	Prp43	ddx-15	CG11107	DHX15	DHX15
						DHX32	DHX32
		UMAG_00419	Dhr2	let-355			DHX33
		UMAG_11913		ddx-35			DHX35
		UMAG_00574			CG9323	DHX29	DHX29
					CG1582	DHX57	DHX57
		UMAG_11114	YLR419W				
		UMAG_05767		rha-1	mle	DHX9	DHX9
							DHX30
						DHX36	DHX36
		UMAG_04665	Dhr1	rha-2	kurz	DHX37	DHX37
				smgl-2	CG32533	DHX34	DHX34
						DHX40	DHX40
	Ski2-like	UMAG_00393	Ski2	skih-2	tst	SkiV2l	SKIV2L
		UMAG_11667	Mtr4	mtr-4	mtr4	SKIV2L2	MTREX
		UMAG_03738	Brr2	snrp-200	CG5931	Snrnp200	SNRNP200
		UMAG_00282	Slh1	Y54E2A.4	obe	Ascc3	ASCC3
		UMAG_04997	Suv3	C08F8.2	Suv3	Supv3l1	SUPV3L1
	RIG-I-like			C28H8.3		DDX60	DDX60
				dcr-1	dcr1	Dicer1	DICER1
				drh-1		DDX58	DDX58
						Ifih1	IFIH1
							DHX58

There are four SF1 Upf1-like RNA helicases in *S. cerevisiae* and eight in *H. sapiens* (Table 1). Our analysis identified four Upf1-like RNA helicases in *U. maydis*. There are three putative orthologs of the characterized SF1 RNA helicases Sen1 (UMAG_10602), Upf1 (UMAG_11428), and Hcs1 (UMAG_01122). Phylogenetic analysis identified the remaining RNA helicase as a putative basidiomycete-specific SF1 RNA helicase (UMAG_10130) (Fig. 2).

Of the 42 SF2 RNA helicases, our investigation identified 27 *U. maydis*DEAD-box RNA helicases (Table 1), of which 25 are conserved and have orthologs in other eukaryotes (Fig. 3). In *S. cerevisiae*, there are 26 DEAD-box RNA helicases, compared to 33 in *H. sapiens*. The remaining two DEAD-box RNA helicases in *U. maydis* (UMAG_05873 and UMAG_00835) are basidiomycete-specific (Fig. 3). Many of these RNA helicases function in a specific aspect of RNA metabolism; however, a few are involved in multiple areas of RNA metabolism.

A total of 10 DEAH/RHA RNA helicases were identified in *U. maydis* based on our sequence (Table 1) and phylogenetic (Fig. 4) analyses, compared to seven in *S. cerevisiae* and 15 in *H. sapiens*. Of the 10, six are spliceosomal DEAH RNA helicases, and the remaining four are RHA-group RNA helicases. The phylogenetic tree (Fig. 4) suggests the presence of a fungal-specific DEAH/RHA RNA helicase with no orthologs in the eukaryotes included in our phylogenetic analysis. This fungal-specific RNA helicase was identified as YLR419W in *S. cerevisiae* and UMAG_11114 in *U. maydis*.

The last family of SF2 RNA helicases is the Ski2-like RNA helicases. Data from *S. cerevisiae* identifies four Ski2-like SF2 RNA helicases: Brr2, Slh1, Mtr4, and Ski2. Suv3 was originally categorized as Ski2-like but has since been removed following further structural analysis (reviewed in Johnson and Jackson 2013). *Ustilago maydis* has orthologs to all five Ski2-like RNA helicases (Fig. 5).

## ﻿Functions of RNA helicases and their potential roles in *Ustilago maydis*

RNA helicases function in all aspects of RNA metabolism, with demonstrated roles in transcription, pre-mRNA splicing, ribosome biogenesis, RNA export, translation, and RNA degradation (Bleichert and Baserga 2007; Jankowsky 2011; Linder and Jankowsky 2011). Despite their broad roles in RNA metabolism, some RNA helicases have specific functions, while others are involved in multiple processes (Jankowsky 2011). Several studies in *H. sapiens* show that upregulation or downregulation of RNA helicases contributes to the development of various diseases and different forms of cancer. Other studies suggest that RNA helicases could serve as therapeutic targets for drug delivery or as diagnostic markers (Shadrick et al. 2013; Zhang and Li 2021). In fungi, RNA helicases have been shown to be important for cellular growth, metabolism, virulence, and stress response (Cheng et al. 2005; Panepinto et al. 2005; Beck et al. 2014; Lauinger et al. 2014; Li et al. 2022; Ying et al. 2022; Paul et al. 2025). Based on current research, we predict that RNA helicases may have additional roles in fungal plant pathology.

A STRING analysis was conducted for each *U. maydis* gene to predict protein-protein interactions (Suppl. material 2: table S1) and infer potential functions. The STRING database (v. 12.0) contains known and predicted protein interactions, and a confidence score is calculated based on supporting evidence from other species (Szklarczyk et al. 2023). We also performed a gene transcript analysis using transcriptomic data from *U. maydis* haploid and dikaryon cultures, as well as dormant and germinating teliospores (Donaldson et al. 2017; Seto et al. 2025), along with transcript patterns during *U. maydis* pathogenesis (Lanver et al. 2018). This analysis allowed us to make predictions about the possible roles RNA helicases may have in *U. maydis* growth, development, and pathogenesis.

The analysis of in planta, biotrophic growth transcript data from Lanver et al. (2018) is summarized in Table 2. Lanver et al. (2018) identified 14 modules, representing groups of co-expressed genes during *U. maydis* pathogenesis. We found that *U. maydis* RNA helicases were present in nine of these modules. A description of the nine expression profiles is summarized in Table 2. Most RNA helicases were found in the green-yellow and yellow modules, indicating that their activity may be important during early stages of biotrophic development and during metabolic and cellular shifts associated with pathogenesis. Transcriptome data from Donaldson et al. (2017) and Seto et al. (2025) were used to determine whether RNA helicase transcript levels were enhanced in the *U. maydis* haploid, dikaryon, or teliospore. RNA helicases with distinct transcript patterns at specific stages of the fungal life cycle are of particular interest. For example, a subset of RNA helicases was found to be upregulated in the dormant teliospore compared to haploid and dikaryon cell types, with distinct changes in transcript levels during teliospore germination. These RNA helicases may be stored in the dormant teliospore or may play roles in stabilizing mRNAs or translating mRNAs during germination (Seto et al. 2025).

**Table 2.** *Ustilago maydis* RNA helicase gene transcript levels during in planta pathogenesis. The modules containing the RNA helicases were identified from the transcriptomic data of Lanver et al. (2018).

UMAG ID	RNA helicase ortholog	Function	Lanver et al. (2018) module	Expression module description
UMAG_10666	Prp28/DDX23	Pre-mRNA splicing	burlywood	Increased at 0.5 dpi, decreases at 2 dpi, and maintained expression
UMAG_10602	Sen1/SETX	Transcription	cyan	Tumor module – increased and maintained expression after biotrophic establishment
UMAG_11428	Upf1	RNA degradation	cyan	
UMAG_01122	Hcs1/IGHMBP2	Translation	cyan	
UMAG_01174	Prp5/DDX46	Pre-mRNA splicing	cyan	
UMAG_04587	DDX41	Pre-mRNA splicing	cyan	
UMAG_00921	Dbp6/DDX51	Ribosome biogenesis	cyan	
UMAG_03268	Spb4/DDX55	Ribosome biogenesis	green	Increases until 1 dpi, decreased and maintained expression between 2–12 dpi
UMAG_05214	Rrp3/DDX47	Ribosome biogenesis	green	
UMAG_11281	Prp43/DHX15	Pre-mRNA splicing, ribosome biogenesis	green	
UMAG_10410	Has1/DDX18	Ribosome biogenesis	green-yellow	Expression peaks at 2 dpi, followed by decrease and is maintained
UMAG_11989	Dbp4/DDX10	Ribosome biogenesis	green-yellow	
UMAG_06228	Dbp7/DDX31	Ribosome biogenesis	green-yellow	
UMAG_10241	Dbp8/DDX49	Ribosome biogenesis	green-yellow	
UMAG_03170	Drs1/DDX27	Ribosome biogenesis	green-yellow	
UMAG_10095	Dbp2/DDX5/DDX17	Transcription, pre-mRNA splicing, ribosome biogenesis, RNA export, RNA degradation	green-yellow	
UMAG_03892	Dbp9/DDX56	Ribosome biogenesis	green-yellow	
UMAG_00242	Mak5/DDX24	Ribosome biogenesis	green-yellow	
UMAG_10683	Rok1/DDX52	Ribosome biogenesis	green-yellow	
UMAG_04665	Dhr1/DHX37	Ribosome biogenesis	green-yellow	
UMAG_11667	Mtr4/MTREX	Ribosome biogenesis, RNA degradation	green-yellow	
UMAG_00835	Unknown	Unknown	light-cyan	Expression decreases at 2 dpi followed by increase at 4 dpi, and is maintained
UMAG_10915	Prp2/DHX16	Pre-mRNA splicing	light-cyan	
UMAG_04188	Prp16/DHX38	Pre-mRNA splicing	light-cyan	
UMAG_03936	Prp22/DHX8	Pre-mRNA splicing	light-cyan	
UMAG_11913	DHX35	Pre-mRNA splicing	light-cyan	
UMAG_03738	Brr2/SNRNP200	Pre-mRNA splicing	light-green	Expressed during the early stages of pathogenesis, followed by decrease
UMAG_00282	Slh1/HELC1	Translation	light-green	
UMAG_10130	Unknown	Unknown	magenta	Increase in expression between 0.5–2 dpi and is maintained
UMAG_05873	Unknown	Unknown	magenta	
UMAG_05200	Dbp10/DDX54	Ribosome biogenesis, pre-mRNA splicing	magenta	
UMAG_04080	Ded1/DDX3	Pre-mRNA splicing, ribosome biogenesis, translation,	magenta	
UMAG_05767	DHX9	Transcription, pre-mRNA splicing, translation	magenta	
UMAG_06129	Fal1/DDX48	Pre-mRNA splicing, ribosome biogenesis, translation	salmon	Decreased during early stages followed by increase after 2 dpi
UMAG_05482	eIF4A/DDX2	Translation initiation	yellow	Increase in expression and peaks at 2 dpi followed by a decreased expression that is maintained
UMAG_10655	Dhh1/DDX6	Translation, RNA degradation	yellow	
UMAG_03765	Dbp5/DDX19	RNA export, translation	yellow	
UMAG_11769	Sub2/DDX39	Pre-mRNA splicing, RNA export	yellow	
UMAG_01732	Dbp3	Ribosome biogenesis	yellow	
UMAG_00652	Mss116	Mitochondrial RNA processing	yellow	
UMAG_06314	Mss116	Mitochondrial RNA processing	yellow	
UMAG_00574	DHX29/DHX57	Translation	yellow	
UMAG_11114	YLR419W	Unknown	yellow	
UMAG_00419	Dhr2/DHX32	Ribosome biogenesis	yellow	
UMAG_00393	Ski2/SkiV2	RNA degradation	yellow	
UMAG_04997	Suv3/SUV3	Mitochondrial RNA processing	yellow	

In the following sections, we highlight the functions of RNA helicases in RNA metabolism. We then utilize STRING and gene transcript analyses to identify specific RNA helicases with potential roles in the biology of phytopathogenic fungi. This has allowed us to identify 28 RNA helicases that may influence *U. maydis* growth, pathogenesis, stress response, and/or teliospore dormancy and germination.

## ﻿Transcription

Transcription is divided into three basic steps: initiation, elongation, and termination. Initiation begins when RNA polymerase recognizes the promoter sequence located upstream of the coding region on a DNA template. Elongation involves the synthesis of the RNA transcript until RNA polymerase encounters the termination sequence. Termination is the dissociation of RNA polymerase from the DNA template and the release of the RNA transcript. During transcription, the nascent RNA transcript undergoes folding, and the speed of transcription elongation can vary, which facilitates interactions with RNA-binding proteins and RNA helicases that may influence RNA folding (reviewed in Pan and Sosnick 2006). Six RNA helicases are known to function during transcription and are summarized in Table 3. *Ustilago maydis* RNA helicases with predicted roles in transcription are orthologs of the *S. cerevisiae* RNA helicases Sen1 and Dbp2 and the *H. sapiens* RNA helicases DHX9, DDX23, and DDX39.

**Table 3.** Functions of RNA helicases during RNA transcription. Orthologs for *U. maydis*, *S. cerevisiae*, and *H. sapiens* are listed.

Superfamily	Family	RNA helicase orthologs			RNA helicase function during transcription	References
		*U. maydis*	*S. cerevisiae*	*H. sapiens*		
SF1	Upf1-like	UMAG_10602	Sen1	SETX	Regulation of R-loops formation and genome stability; Sen1-dependent pathway to aid in RNA polymerase dissociation from DNA template	Mischo et al. (2011); Hazelbaker et al. (2013); Groh et al. (2017); Hegazy et al. (2020); Aiello et al. (2022)
SF2	DEAD-box	UMAG_10666	Prp28	DDX23	DDX23 suppresses R-loop accumulation by removing RNA polymerase II during transcription elongation	Sridhara et al. (2017)
		UMAG_11769	Sub2	DDX39B	DDX39B removes unscheduled R-loops during transcription by unwinding RNA:DNA hybrids	Pérez-Calero et al. (2020)
		UMAG_10410	Has1	DDX18	Interacts with PARP-1 and R-loops to mediate DNA damage and maintain genome stability	Lin et al. (2022)
		UMAG_10095	Dbp2	DDX5 DDX17	A dsRNA-dependent ATPase that is recruited to the chromatin to facilitate RNA structural rearrangements	Cloutier et al. (2012); Ma et al. (2013); Ma et al. (2016); Xing et al. (2019)
	DEAH/RHA	UMAG_05767		DHX9	DHX9 with PARP1 to maintain genome stability and prevent DNA damage caused by R-loop formation	Cristini et al. (2018)

UMAG_10602 was identified as an RNA helicase that may function during pathogenic development. It is the ortholog of the SF1 Upf1-like RNA helicase Sen1 in *S. cerevisiae* and SETX in *H. sapiens* (Fig. 2; Table 1). STRING analysis predicts a conserved role in transcription. Depletion or mutation of SETX has been found to affect the expression of a subset of genes involved in cytoskeletal organization, autophagy regulation, and lysosomal degradation (Richard et al. 2021; Giannini and Porrua 2024). Transcriptome analysis of data from Donaldson et al. (2017) and Seto et al. (2025) indicates that the gene transcript is present in all *U. maydis* cell types, including during teliospore dormancy and germination. During *U. maydis* infection, the transcript is present in the cyan/tumor module (Table 2). Genes in this module are induced following biotrophic establishment and at the onset of tumor formation (Lanver et al. 2018). This transcript pattern for UMAG_10602 suggests a role in maintaining genome integrity during the transcription of genes required for tumor growth and development. We predict that UMAG_10602 mutants may affect pathogenic development and result in decreased pathogenesis.

## ﻿Pre-mRNA splicing

Eukaryotic precursor messenger RNA (pre-mRNA) is subjected to splicing before it becomes a functional mRNA. This process is highly regulated and involves two consecutive transesterification steps catalyzed by the spliceosome. Regulation and efficiency of splicing ensure the proper removal of introns and fusion of exons to form mature mRNA. During splicing, the spliceosome undergoes structural rearrangements mediated by RNA helicases. The identified *U. maydis* helicases predicted to be involved in these processes are listed in Table 4. Most of these RNA helicases act as chaperones that modulate the structure of the mRNA or a ribonucleoprotein (RNP) associated with the mRNA (Liu and Cheng 2015). A total of eight RNA helicases are required for splicing in *S. cerevisiae*: Sub2, Prp5, Prp28, Brr2, Prp2, Prp16, Prp22, and Prp43. In general, these helicases act as chaperones that alter the structure of RNA or RNPs (Liu and Cheng 2015), and their specific functions are listed in Table 4. Other RNA helicases that may function during pre-mRNA splicing include Dbp2 and Ded1 in *S. cerevisiae* and DHX9, DHX35, DDX41, DDX48, and DDX54 in *H. sapiens*. The *U. maydis* orthologs of these proteins, along with summaries of their predicted functions, are provided in Table 4.

**Table 4.** Functions of RNA helicases during pre-mRNA splicing. Orthologs for *U. maydis*, *S. cerevisiae*, and *H. sapiens* are listed.

Superfamily	Family	RNA helicase orthologs			RNA helicase function during pre-mRNA splicing	References
		*U. maydis*	*S. cerevisiae*	*H. sapiens*		
SF2	DEAD-box	UMAG_11769	Sub2	DDX39A DDX39B	Responsible for the displacement and recruitment of specific RNPs at the pre-mRNA intron branch site; DDX39A and DDX39B regulate alternative splicing	Luo et al. (2001); Liu and Cheng (2015); Banerjee et al. (2024)
		UMAG_01174	Prp5	DDX46	Interacts with snRNP U2 to mediate conformation changes and stabilization; Release of Prp5 signals the recruitment of the U4/U6.U5 tri-snRNP; DDX46 induces pre-mRNA conformational changes for splicing	Liu et al. (2007); Liang and Cheng (2015); Bourgeois et al. (2016)
		UMAG_10666	Prp28	DDX23	Destabilizes the interaction between snRNP U1 and the 5’ splice site and allows for U6 to bind to the site; May proofread the 5’ splice site during spliceosome assembly	Liu and Cheng (2015); Bourgeois et al. (2016)
		UMAG_04188	Prp16	DDX38	Induces spliceosome conformational changes before initiation of the second catalytic reaction; Displaces proteins associated with the spliceosome causing the catalytic core to be less rigid to allow for contact with the 3’ splice site; Prp16 functions with Prp22 to remove stalled spliceosomes and repress suboptimal splicing sites to allow for alternative splice site selection	Liu and Cheng (2015); Robert-Paganin et al. (2015); Bourgeois et al. (2016); Semlow et al. (2016)
		UMAG_10095	Dbp2	DDX5 DDX17	DDX5 mediates the interaction between U1 and the 5’ splice site; Serves as a bridge for communication between the splice site and the U4/U6.U5 tri-snRNP DDX17 assists the U1 in recognizing the 5’ splice site and facilitates alternative splicing in select human genes	Hönig et al. (2002); Lee (2002); Liu (2002)
		UMAG_04080	Ded1 Dbp1	DDX3	Interacts with the spliceosome however exact role is not fully understood	Jamieson et al. (1991)
		UMAG_04587		DDX41	DDX41 and the *C. elegans* ortholog sacy-1 interact with spliceosome components and can affect alternative splicing	Polprasert et al. (2015); Tsukamoto et al. (2020); Andreou (2021)
		UMAG_06129	Fal1	DDX48	DDX48 facilitates the assembly of the exon junction complex	Linder and Jankowsky (2011)
		UMAG_5200	Dbp10	DDX54	DDX54 binds to introns that contain a weak 3’ splice site to increase the splicing rate	Rodríguez-Galán et al. (2013); Milek et al. (2017)
	DEAH/RHA	UMAG_10915	Prp2	DHX16	Remodels the spliceosome into its catalytically active form with cofactor Spp2; ATPase activity destabilizes the snRNPU2 from the spliceosome to expose the branchpoint	Liu and Cheng (2015); Bourgeois et al. (2016)
		UMAG_03936	Prp22	DHX8	Facilitates the release of mature mRNA by disrupting RNA-RNA or RNA-protein interactions; Capable of discarding aberrant spliceosomes from pre-mRNA; Prp22 and Prp16 function together to repress suboptimal splicing sites and aid in alternative splice site selection	Robert-Paganin et al. (2015); Semlow et al. (2016)
		UMAG_11281	Prp43	DHX15	Mediates disassembly of the spliceosome after splicing completion or removal of an impaired or arrested spliceosome	Liu and Cheng (2015)
SF2	DEAH/RHA	UMAG_05767		DHX9	Binds to pre-mRNA and snRNPs that are components of the splicing machinery suggesting a possible role during splicing	Lee and Pelletier (2016)
		UMAG_11913		DHX35	DHX35 interacts with other spliceosome proteins	Sales-Lee et al. (2021)
	Ski2-like	UMAG_03738	Brr2	SNRNP200	Brr2 is a component of the snRNP U5 and mediates the unwinding of U4/U6 to release U4 during spliceosome activation; Regulates other splicing factors by serving as a platform for their recruitment; Promotes protein-protein interactions; Capable of inducing structural changes to the pre-mRNA	Liu and Cheng (2015)

The splicing pathway begins with the assembly of the spliceosome, which is initiated by recognition and binding of snRNA U1 at the 5′ splice site. This results in the formation of commitment complex 1 (CC1) and the prespliceosome, where snRNA U2 binds to the branch point sequence (Liu and Cheng 2015). The two DEAD-box RNA helicases involved in this process are Sub2 and Prp5 in *S. cerevisiae*, and their *H. sapiens* orthologs are DDX39 and DDX46 (Bleichert and Baserga 2007; Liu and Cheng 2015). The *U. maydis* orthologs of these helicases are UMAG_11769 and UMAG_01174. There is evidence that DDX5 and DDX17 may assist this process in *H. sapiens* (Hönig et al. 2002; Lee 2002), and the *U. maydis* ortholog to these proteins is UMAG_10095.

The second step of the splicing pathway is spliceosome activation, which involves recruitment of the U4/U6.U5 tri-snRNP, and the pre-mRNA undergoes additional conformational changes. These structural changes and snRNP binding result in a stable structure that forms the basis of the catalytic core of the spliceosome (Liu and Cheng 2015). The DEAD-box RNA helicase Prp28 in *S. cerevisiae*, DDX23 in *H. sapiens*, the *U. maydis* ortholog UMAG_10666, and the Ski2-like RNA helicase Brr2 in *S. cerevisiae* and *U. maydis* ortholog UMAG_03738 modulate this process (Liu and Cheng 2015; Bourgeois et al. 2016).

Two catalytic reactions occur after spliceosome activation. These reactions require the function of two RNA helicases: the SF2 DEAH RNA helicase Prp2 and the SF2DEAD-box RNA helicase Prp16 in *S. cerevisiae*, DHX16 and DDX38 in *H. sapiens* (Liu and Cheng 2015; Bourgeois et al. 2016), and *U. maydis* orthologs UMAG_10095 and UMAG_04188. After the completion of mRNA splicing, the spliceosome is disassembled, and mature mRNA is released. Two SF2 DEAH RNA helicases are required for this step: Prp22 and Prp43 in *S. cerevisiae*, and DHX8 and DHX15 in *H. sapiens* (Liu and Cheng 2015; Bourgeois et al. 2016). The *U. maydis* orthologs to these proteins are UMAG_03936 and UMAG_11281. It is notable that some helicases have more than one role in pre-mRNA splicing.

The transcript analysis of the *U. maydis* RNA helicases predicted to be involved in pre-mRNA splicing revealed five RNA helicases that may modulate pathogenesis, growth, and teliospore formation and germination. These RNA helicases are UMAG_04587, UMAG_10666, UMAG_01174, UMAG_11913, and UMAG_06129. The transcripts of these RNA helicases are found in the haploid and dikaryon cell types, as well as during teliospore dormancy and germination (Donaldson et al. 2017; Seto et al. 2025). The RNA helicases UMAG_04587 and UMAG_01174 are found in the Lanver et al. (2018) cyan module, representing the tumor development module. UMAG_10666 is the only RNA helicase found in the burlywood module, and UMAG_11913 is found in the light-cyan module (Table 2). The UMAG_06129 transcript is upregulated in the dormant teliospore and remains unchanged during germination (Seto et al. 2025). It is the only RNA helicase in the Lanver et al. (2018) salmon module (Table 2).

UMAG_04587 is a putative RNA helicase with orthologs in basidiomycete fungi and animals; however, no orthologs were identified in the ascomycete fungi included in our analysis (Fig. 3). STRING analysis suggests that UMAG_04587 interacts with proteins involved in pre-mRNA splicing (Suppl. material 2: table S1), consistent with other DDX41 orthologs. In *H. sapiens*, DDX41 may modulate the expression of a subset of proteins at the posttranslational level (Peters et al. 2017). In *D. melanogaster*, the ortholog Abstrakt is involved in the posttranscriptional regulation of the gene *Insc* by binding to its mRNA. Loss of Abstrakt results in decreased *Insc* protein levels and defects in cell polarity and division (Irion et al. 2004). DDX41 also plays a role in the innate immune response and acts as an antiviral factor against viral infections by sensing viral DNA and DNA:RNA duplexes, thereby modulating nucleic acids to trigger a host immune response (Li et al. 2018; Soponpong et al. 2018; Liu et al. 2019; Winstone et al. 2024). If UMAG_04587 functions as a viral sensor, it may contribute to detecting and responding to mycovirus infections. The presence of its transcript in the cyan/tumor module during pathogenesis (Lanver et al. 2018; Table 2) suggests that UMAG_04587 may play an enhanced role during the onset of tumor formation *in planta*, possibly by modulating gene expression at the posttranscriptional or posttranslational level. We hypothesize that if UMAG_04587 regulates cell polarity during growth, deletion mutants will exhibit growth defects.

STRING analysis predicts that UMAG_10666 interacts with other spliceosomal proteins, suggesting a functional role in pre-mRNA splicing in *U. maydis* (Suppl. material 2: table S1). This RNA helicase is the ortholog of Prp28 in *S. cerevisiae* and DDX23 in *H. sapiens* (Fig. 3 and Table 1). The *C. elegans* ortholog DDX-23 is required for embryonic and postembryonic development and cell differentiation, possibly by modulating specific RNA-protein interactions (Konishi et al. 2008; Chu et al. 2016). If this function is conserved, UMAG_10666 may regulate a specific subset of genes during key developmental transitions. Its transcript profile during pathogenesis suggests that elevated levels of UMAG_10666 may be required for splicing transcripts involved in the early stages of pathogenesis and/or in modulating RNA-protein interactions and splicing of genes that drive tumor formation.

UMAG_01174 was identified as the putative ortholog to Prp5/DDX46 (Fig. 3). STRING analysis predicts that UMAG_01174 interacts with proteins involved in pre-mRNA splicing (Suppl. material 2: table S1). Interestingly, STRING also predicts an interaction with the uncharacterized protein UMAG_11076. This protein is the putative ortholog of the *S. cerevisiae* protein Urn1, which plays a role in cell cycle progression (Niu et al. 2008). Similarly, DDX46 has been shown to contribute to cell proliferation and cell cycle progression pathways (Li et al. 2016). If the role of this RNA helicase is conserved across species, UMAG_01174 may contribute to cell cycle progression and proliferation in *U. maydis* cells. This helicase is also present in the cyan/tumor module during pathogenesis (Lanver et al. 2018) (Table 2), suggesting that its activity may modulate the splicing of transcripts encoding proteins or directly affect proteins that contribute to *U. maydis* growth and tumor induction.

The putative SF2 RNA helicase UMAG_11913 was identified as the ortholog of DHX35 in *H. sapiens* (Fig. 4, Table 1). STRING analysis suggests that UMAG_11913 interacts with other spliceosomal proteins, further supporting a conserved function across species (Suppl. material 2: table S1). Functional characterization of UMAG_11913 may confirm its role in the *U. maydis* spliceosome. In *Magnaporthe oryzae*, another fungal plant pathogen, MoDHX35 deletion mutants exhibit reduced appressorium formation and decreased virulence (Ying et al. 2022). The UMAG_11913 transcript is found in the light-cyan module during *U. maydis* pathogenesis, indicating that it is upregulated during the early stages of infection, followed by a decrease and then an increase (Lanver et al. 2018). We hypothesize that if the molecular regulation of UMAG_11913 mirrors that of MoDHX35, appressorium formation in *U. maydis* may be impaired, leading to reduced pathogenicity. Deletion strains of UMAG_11913 should be generated to assess whether a shared phenotype exists with *M. oryzae*. A similar phenotype would suggest a conserved function for this RNA helicase in fungal plant pathogens.

Fal1, the budding yeast ortholog of UMAG_06129, is essential in *S. cerevisiae*, whereas deletion mutants are viable in fission yeast (Kressler et al. 1997; Kim et al. 2010). Our STRING analysis of UMAG_06129 (Suppl. material 2: table S1) predicts protein-protein interactions with components of the splicing machinery and ribosome biogenesis. Interestingly, *S. pombe* Fal1 interacts with Red5, a subunit of the MTREC complex, to regulate the splicing of meiosis transcripts. These transcripts are unspliced and targeted for degradation during mitosis. The interaction between Fal1 and Red5 enables splicing of meiosis-specific genes during meiosis (Marayati et al. 2016). The *U. maydis* genome contains orthologs of Red5, most MTREC complex components, and the EJC, suggesting that UMAG_06129 may function similarly by identifying meiosis transcripts and facilitating their translation during meiosis. The UMAG_06129 transcript is upregulated in the dormant teliospore compared to the haploid and dikaryon cell types (Seto et al. 2025). It is the only RNA helicase in the salmon module, which includes genes upregulated after tumor formation and during teliospore development (Table 2) (Lanver et al. 2018). This expression pattern suggests a role in the regulation of genes associated with teliospore development, dormancy entry, and meiotic pause. UMAG_06129 may contribute to the translation of these genes and the early meiotic processes before teliospore dormancy. Functional characterization of UMAG_06129 will clarify its role in *U. maydis*. If it functions similarly to its *S. pombe* ortholog, it may influence teliospore formation and germination, processes that coincide with meiosis in *U. maydis*.

## ﻿RNA export

After splicing, the mRNA undergoes additional modifications and binds various proteins to become competent for nuclear export. This process requires the recruitment of multiple adaptor proteins, which depend on RNA helicases to facilitate recruitment, protein dissociation, or mRNA remodeling in both the nucleus and cytoplasm (Iglesias and Stutz 2008; Bourgeois et al. 2016). The RNA helicases Sub2, Dbp2, and Dbp5 in *S. cerevisiae* have been identified as playing roles in RNA export from the nucleus, and their *U. maydis* orthologs are UMAG_11769, UMAG_10095, and UMAG_03765, respectively (Table 5).

**Table 5.** Functions of RNA helicases during RNA export. Orthologs in *U. maydis*, *S. cerevisiae*, and *H. sapiens* are listed.

Superfamily	Family	RNA helicase orthologs			RNA helicase function during RNA export	References
		*U. maydis*	*S. cerevisiae*	*H. sapiens*		
SF2	DEAD-box	UMAG_11769	Sub2	DDX39A DDX39B	Responsible for recruiting the protein Yra1 to the mRNA and is then displaced to allow the mRNP to dock onto the nuclear pore complex	Luo et al. (2001); Sträßer and Hurt (2001); Noble et al. (2011)
		UMAG_10095	Dbp2	DDX5 DDX17	Aids in the assembly of Yra1, Nab2, and Mex67 on the poly(A)+ tail by unwinding and remodelling the mRNA duplex	Ma et al. (2013); Xing et al. (2019)
		UMAG_03765	Dbp5	DDX19A DDX19B DDX25	Dbp5 initiates mRNA remodelling when an mRNP is exported through the nuclear pore	Tran et al. (2007); Noble et al. (2011)

Transcriptome analysis of the three *U. maydis* RNA helicases predicted to be involved in RNA export revealed that both UMAG_11769 and UMAG_03765 have transcript levels that are not significantly different between cell types or during teliospore dormancy and germination (Donaldson et al. 2017; Seto et al. 2025). Both transcripts are also found in the yellow module during pathogenesis, indicating co-expression with genes involved in cellular metabolism (Table 2) (Lanver et al. 2018). The UMAG_10095 transcript was found to be upregulated in the dormant teliospore and remained unchanged during teliospore germination (Seto et al. 2025). During pathogenesis, UMAG_10095 is present in the green-yellow module (Table 2) (Lanver et al. 2018), representing gene transcripts that increase and peak at 2 dpi before decreasing during the late stages of pathogenesis. UMAG_11769 and UMAG_10095 were identified as having possible roles in *U. maydis* growth, pathogenesis, and regulation of teliospore dormancy and germination.

UMAG_11769 was previously described by Feldbrügge et al. (2008) as the putative ortholog to the *S. cerevisiae* RNA helicase Sub2 and *H. sapiens* DDX39A/DDX39B (Fig. 3, Table 1). STRING analysis predicts protein-protein interactions with proteins involved in spliceosome assembly and the export of polyadenylated mRNAs (Suppl. material 2: table S1), suggesting a conserved function across eukaryotes. The *Danio rerio* ortholog, Ddx39ab, is required for the development of the heart, trunk muscles, and eyes during embryogenesis. This helicase binds to a specific subset of epigenetic regulatory factors involved in the development of these tissues. Loss of *ddx39ab* results in abnormal pre-mRNA splicing and misregulation of developmental genes (Zhang et al. 2018). The *H. sapiens* orthologs DDX39A and DDX39B (Fig. 3, Table 1) are components of the mRNA export machinery and are linked to mitotic progression. It has been proposed that these helicases regulate gene expression of mitosis-related genes, with each helicase targeting different gene sets (Yamazaki et al. 2010). If UMAG_11769 functions similarly, we predict that deletion mutants will exhibit growth and mitotic defects.

STRING analysis for the Dbp2 ortholog UMAG_10095 predicts interactions with proteins involved in transcription, splicing, ribosome biogenesis, RNA export, and RNA degradation (Suppl. material 2: table S1), supporting a conserved function. In yeast and mammals, Dbp2/DDX5 is linked to maintaining cellular homeostasis. It plays a role in glucose sensing and promoting glycolysis during growth (Beck et al. 2014; Xing et al. 2017). More recently, Dbp2 has been shown to modulate the stress response to low glucose by binding specific mRNAs, retaining them in the nucleus, and promoting their degradation under nutrient-rich conditions. Under nutrient-poor conditions, Dbp2 relocates to the cytoplasm to facilitate export of these mRNAs for translation (Paul et al. 2025). We previously hypothesized that upregulation of UMAG_10095 in dormant and germinating teliospores enables *U. maydis* to detect changes in glucose availability and coordinate RNA processing accordingly (Seto et al. 2025). *DBP2* is nonessential in *S. cerevisiae* (Bond et al. 2001; Cloutier et al. 2012), and we predict that UMAG_10095 deletion will be nonlethal in *U. maydis*, but deletion or overexpression mutants will likely exhibit a slow growth phenotype, as observed in *S. cerevisiae DBP2* mutants (Cloutier et al. 2012). Additionally, UMAG_10095 deletion mutants may be impaired in teliospore germination.

## ﻿Ribosome biogenesis

Approximately 20 different RNA helicases are involved in ribosome biogenesis, with each helicase performing a specific function during the process. The *U. maydis* orthologs of these RNA helicases are listed in Table 6. The first step of ribosome biogenesis is the transcription of rRNA. The large primary rRNA transcript—called the 35S pre-rRNA in *S. cerevisiae*—is transcribed by RNA polymerase I and contains the 18S, 5.8S, and 25S/28S rRNAs. The 5S rRNA is transcribed separately by RNA polymerase III. Processing of the pre-rRNA involves cleavage at specific sites in the external and internal transcribed spacers (ETS and ITS) by endonucleases and exonucleases, producing mature rRNAs (Martin et al. 2013; Rodríguez-Galán et al. 2013).

**Table 6.** Functions of RNA helicases during ribosome biogenesis. Orthologs in *U. maydis*, *S. cerevisiae*, and *H. sapiens* are listed.

Superfamily	Family	RNA helicase orthologs			RNA helicase function during ribosome biogenesis	References
		*U. maydis*	*S. cerevisiae*	*H. sapiens*		
SF2	DEAD-box	UMAG_10095	Dbp2	DDX5 DDX17	Dbp2 facilitates structural rearrangements of the pre-rRNA; DDX5 and DDX17 are capable of displacing snoRNA U8 from the pre-rRNA	Bond et al. (2001); Jalal et al. (2007)
		UMAG_01732	Dbp3		Facilitate access to the A_3_ cleavage site of ITS1 in the 27S A2 pre-rRNA for the RNase MRP complex	Weaver et al. (1997)
		UMAG_00921	Dbp6	DDX51	Dbp6 facilitates structural rearrangements of the pre-rRNA to allow for efficient assembly of the 60S ribosomal subunit DDX51 promotes the displacement of proteins during the processing of the 3’ end of the 28S rRNA	Kressler et al. (1998); Srivastava et al. (2010); Martin et al. (2013); Rodríguez-Galán et al. (2013)
		UMAG_06228	Dbp7	DDX31	Dbp7 remodels the 35S pre-rRNA which allows for the attachment and release of proteins during the maturation of the pre-rRNA	Fukawa et al. (2012); Aquino et al. (2021)
					DDX31 regulates rRNA transcription	
		UMAG_03892	Dbp9	DDX56	May modulate pre-rRNA rearrangements during 18S rRNA maturation	Zirwes et al. (2000); Daugeron et al. (2001); Rodríguez-Galán et al. (2013)
					DDX56 may function during the maturation of pre-rRNA during maturation of the 60S ribosomal subunit	
		UMAG_5200	Dbp10	DDX54	Bind to pre-rRNA to induce conformational changes or act as a chaperone for other proteins to facilitate cleavage	Burger et al. (2000); Rodríguez-Galán et al. (2013); Mitterer et al. (2023)
		UMAG_03170	Drs1	DDX27	Drs1 is tightly associated with Dbp6, Dbp7, Dbp9, Mak5, and Has1 during synthesis of the 60S ribosomal subunit DDX27 associates with pre-rRNA to recruit the protein complex PeBoW	Ripmaster et al. (1992); Bernstein et al. (2006); Martin et al. (2013); Kellner et al. (2015)
		UMAG_00242	Mak5	DDX24	Mak5 may function within a protein cluster or creates a stable association between ribosomal proteins during 60S assembly DDX24 interacts with MDM2 to mediate ubiquitylation and degradation of p53 to increase transcription of the 47S transcript	Zagulski et al. (2003); Pratte et al. (2013); Yamauchi et al. (2014)
		UMAG_11667	Mtr4	MTREX	Functions within the TRAMP complex that contributes to the polyadenylation of 3’ ends of snoRNAs and rRNAs by modulating the activity of the TRAMP complex by unwinding the RNA to generate longer stretches of ssRNA; Can impact RNA binding, ATP affinity, rate of adenylation, and TRAMP dissociation	Jia et al. (2011); Martin et al. (2013); Rodríguez-Galán et al. (2013)
		UMAG_03268	Spb4	DDX55	Spb4 interacts with Pwp2 to facilitate the processing of the 35S pre-rRNA during the early and late stages of maturation DDX55 remodels pre-rRNA during the maturation process	Sachs and Davis (1990); de la Cruz et al. (1998a); Dosil and Bustelo (2004); García-Gómez et al. (2011); Choudhury et al. (2021)
		UMAG_11989	Dbp4	DDX10	May facilitate the release of snoRNAs from pre-rRNA	Savitsky et al. (1996); Koš and Tollervey (2005)
SF2	DEAD-box	UMAG_10241	Dbp8	DDX49	Dbp8 is involved in the maturation of the 18S rRNA	Daugeron and Linder (2001); Granneman et al. (2006); Awasthi et al. (2018)
					DDX49 binds to regulatory regions to provide stability to the rRNA	
		UMAG_06129	Fal1	DDX48	Fal1 may function during the maturation of the 18S rRNA	Kressler et al. (1997); Alexandrov et al. (2011); Rodríguez-Galán et al. (2013); Choe et al. (2014)
					DDX48 may remodel or promote unwinding when a secondary structure is encountered	
		UMAG_10683	Rok1	DDX52	Facilitates the release of snoRNAs from pre-rRNA which allows access to cleavage sites	Venema et al. (1997); Bohnsack et al. (2008); Martin et al. (2013); Rodríguez-Galán et al. (2013)
		UMAG_05214	Rrp3	DDX47	Mediates structural changes that allow for snoRNAs to bind and cleavage to the pre-rRNA	O’Day et al. (1996); Sekiguchi et al. (2006); Martin et al. (2013); Rodríguez-Galán et al. (2013)
		UMAG_10410	Has1	DDX18	Coordinates the assembly of proteins on the pre-rRNA to allow for cleavage to occur	Martin et al. (2013); Rodríguez-Galán et al. (2013)
		UMAG_04080	Ded1 Dbp1	DDX3	Protein detected with pre-ribosomal particles, but function is unknown	Krogan et al. (2004); Sharma and Jankowsky (2014)
	DEAH/RHA	UMAG_04665	Dhr1	DHX37	Remodels pre-rRNA for processing and releases U3 to allow for cleavage to occur in the 5’ ETS and ITS1	Colley et al. (2000); Choudhury et al. (2019)
		UMAG_ 00419	Dhr2	DHX32	Dhr2 initiates cleavage at site A_0_ of the 5’ ETS region of the pre-rRNA	Colley et al. (2000); Choque et al. (2011); Martin et al. (2013)
					DHX32 interacts with pre-rRNA processing proteins during rRNA maturation	
		UMAG_11281	Prp43	DHX15	May displace snoRNAs from the pre-rRNA and modulate structural changes to the transcript	Rodríguez-Galán et al. (2013)

The ribosome is composed of the large and small subunits. The large subunit, known as the 60S in eukaryotes, consists of the 25S/28S, 5.8S, and 5S rRNAs and 46 ribosomal proteins (Martin et al. 2013). The SF2DEAD-box RNA helicases involved in processing the 60S subunit in *S. cerevisiae* include Dbp2, Dbp3, Dbp6, Dbp7, Dbp9, Dbp10, Drs1, Mak5, Mtr4, and Spb4. The 40S, the small ribosomal subunit in eukaryotes, comprises the 18S rRNA and 33 other ribosomal proteins. Seven different RNA helicases are involved in processing the 40S subunit: Dbp4, Dbp8, Dhr1, Dhr2, Fal1, Rok1, and Rrp3. Two RNA helicases, Prp43 and Has1, participate in processing both the 60S and 40S subunits (Martin et al. 2013; Rodríguez-Galán et al. 2013). It has also been suggested that Ded1 in *S. cerevisiae* may function during ribosome biogenesis, as the protein has been detected in association with pre-ribosomal particles, although its exact role is not currently understood (Krogan et al. 2004; Sharma and Jankowsky 2014). Table 6 summarizes the specific functions of these RNA helicases during ribosome biogenesis.

Many RNA helicases involved in ribosome biogenesis are essential proteins. We used current knowledge and *U. maydis* transcriptome data to identify nine RNA helicases with potential roles in the *U. maydis* life cycle. The UMAG_11989, UMAG_30170, UMAG_10683, and UMAG_11281 transcripts were not significantly different across all cell types and during teliospore dormancy and germination (Donaldson et al. 2017; Seto et al. 2025). During pathogenesis, these transcripts are found in the green-yellow (UMAG_11989, UMAG_30170, UMAG_10683) and green (UMAG_11281) modules (Table 2). The transcripts for UMAG_10410, UMAG_05214, UMAG_01732, UMAG_10241, and UMAG_05200 were upregulated in the dormant teliospore compared to the haploid and dikaryon cell types (Seto et al. 2025). These transcripts are present in the green (UMAG_05214), green-yellow (UMAG_10410, UMAG_10241), yellow (UMAG_01732), and magenta (UMAG_05200) modules during pathogenesis (Table 2). We predict that these RNA helicases contribute to *U. maydis* growth, pathogenesis, stress response, and teliospore dormancy.

The *U. maydis* ortholog of the *S. cerevisiae* RNA helicase Has1 was identified as UMAG_10410 (Fig. 3, Table 1). Based on STRING analysis, its role in rRNA processing is likely conserved (Suppl. material 2: table S1). Has1 is essential in *S. cerevisiae*, and we predict that deletion of UMAG_10410 will be lethal. In zebrafish, the ortholog DDX18 is involved in cell cycle progression during embryogenesis; loss of this RNA helicase results in cell cycle arrest and disrupted hematopoiesis (Payne et al. 2011). In human lung cancer cells, DDX18 depletion causes cell cycle arrest at the G1 phase, whereas overexpression promotes cell proliferation (Feng et al. 2024). We predict that altering the expression of UMAG_10410 will result in growth defects. Based on the *U. maydis* pathogenesis transcriptome data from Lanver et al. (2018), the peak transcript level of UMAG_10410 at 2 dpi corresponds to the shift to cell proliferation following cell cycle arrest before appressoria penetration. This pattern suggests that UMAG_10410 may play a role during key developmental transitions in *U. maydis* growth within the plant. Additionally, UMAG_10410 is upregulated in the dormant teliospore and remains stably expressed during teliospore germination (Seto et al. 2025). This expression pattern indicates that UMAG_10410 may have an important role during the transition from dormancy to germination, possibly through maintaining genome stability or regulating DNA repair during this developmental switch.

UMAG_11989 is the ortholog to *S. cerevisiae* RNA helicase DBP4 and DDX10 in *H. sapiens*. Overexpression of the *H. sapiens* ortholog DDX10 results in the proliferation of several types of cancer (Yassin et al. 2010; Zhou et al. 2022). In colorectal cancer cells, DDX10 was shown to interact with the ribosomal protein RPL35, which is a component of the 60S subunit. Overexpression of this RNA helicase resulted in alternative splicing of *RPL35* mRNA, which then affected the downstream E2F pathway—a pathway that regulates the cell cycle and cancer development (Zhou et al. 2022). *Ustilago maydis* contains the ortholog to *RPL35* (UMAG_11625), indicating the potential for UMAG_11989 to function similarly. Based on *U. maydis* transcriptome data, UMAG_11989 may have an enhanced role during the early stages of pathogenesis, possibly during the establishment of biotrophic development after the dikaryotic filaments penetrate the plant host. It is hypothesized that it enhances the expression of a subset of genes required for cell cycle progression. If UMAG_11989 regulates the cell cycle through its interaction with other proteins, altering its expression may affect the growth of *U. maydis* cells and impact its development within plant tissue.

In *H. sapiens*, cell homeostasis can be disrupted by unscheduled R-loop formation. The RNA helicase DDX47 resolves R-loop formation by unwinding these DNA:RNA hybrids (Marchena-Cruz et al. 2023). The ortholog of this in *U. maydis* was identified as UMAG_05214 and in *S. cerevisiae* as Rrp3 (Fig. 3, Table 1). We previously identified UMAG_05214 with upregulated transcript levels in the dormant teliospore, which remained at a steady state during germination (Seto et al. 2025). This suggests that UMAG_05214 may have an enhanced role during germination, and we hypothesize that it may function to regulate R-loop formation during transcription. UMAG_05214 may have an additional role in promoting the transcription of genes involved in maintaining cellular homeostasis in other cell types. Disruption in the function of this RNA helicase in *U. maydis* may cause irregular cell growth and dysregulation of cellular metabolism. Functional characterization is required to determine the impact of UMAG_05214 on teliospore germination and *U. maydis* growth.

UMAG_10683 was identified as the putative *U. maydis* Rok1/DDX52 ortholog (Fig. 2, Table 1). Our STRING analysis predicts that UMAG_10683 is involved in processing rRNA, suggesting that its function is conserved. In *C. elegans*, DDX-52 loss-of-function mutants were resistant to hypoxia, experienced arrested early development, and underwent cell death at high temperatures (Itani et al. 2021). In *S. cerevisiae*, Rok1 is an essential protein that functions to regulate cell cycle progression (Song et al. 1995; Giaever et al. 2002; Jeon and Kim 2010). *S. cerevisiae* cells with disrupted or overexpressed Rok1 are arrested at the G1/S phase of the cell cycle. This suggests that the protein levels of Rok1 regulate ribosome biogenesis at the beginning of the cell cycle (Jeon and Kim 2010). We hypothesize that deletion of UMAG_10683 will be lethal. If UMAG_10683 functions similarly to its orthologs, altering its expression will result in defects in cell cycle progression and altered growth rates. During *U. maydis* pathogenesis, the gene transcript increases and peaks at 2 dpi, indicating that enhanced transcription of UMAG_10683 may aid in the translation of genes during this stage of pathogenesis.

In *S. cerevisiae*, RNA helicase Dbp3 null mutants were observed to have increased thermotolerance, oxidative stress resistance, DNA stress, and endoplasmic reticulum stress resistance (Delaney et al. 2013). The *Arabidopsis thaliana* orthologs are negative regulators of stress-responsive transcription activators (Kant et al. 2007; Khan et al. 2014). Overexpression studies of *S. cerevisiae DBP3* suggested a role in protein secretion in fungal cells (Chen et al. 2023). Our phylogenetic and sequence analysis identified UMAG_01732 as the ortholog to *Dbp3* in *S. cerevisiae* and *STRS1* in *A. thaliana*. We did not identify orthologs in any other species included in our analysis (Fig. 3, Table 1). Our STRING analysis predicted interactions with proteins involved in ribosome biogenesis (Suppl. material 2: table S1), suggesting that this RNA helicase has a conserved function in fungi and plants. The UMAG_01732 gene transcript is upregulated in the dormant teliospore and decreases during teliospore germination (Seto et al. 2025). This transcript pattern suggests that this RNA helicase may be involved in the transition from a dormant state to one of high metabolic activity. We had named this RNA helicase *udbp3* and created deletion mutants in the compatible haploid strains, 518 and 521 (Seto and Saville 2025). Deletion mutants were viable and showed no significant difference in dikaryon formation, pathogenesis, or teliospore formation and germination. We found that the deletion mutants were more tolerant to osmotic stress (Seto and Saville 2025). It was concluded that *udbp3* may be a negative regulator of osmotic stress response by regulating a subset of stress-responsive genes during teliospore dormancy (Seto and Saville 2025). Future work should focus on creating overexpression mutants to determine if it functions similarly to *DBP3*. *udbp3* may have a similar role in regulating the protein secretion pathway to ensure the development and growth of the promycelium during teliospore germination.

Our STRING analysis for UMAG_03170 (Suppl. material 2: table S1) predicts that this putative RNA helicase functions during the synthesis of ribosomal subunits, suggesting a conserved role across eukaryotes. Its orthologs, Drs1 in *S. cerevisiae* and DDX27 in *H. sapiens* (Fig. 3, Table 1), have demonstrated roles during ribosome biogenesis (Table 5). The zebrafish ortholog, DDX27, contributes to the processing of ribosomal subunits and was also found to regulate the translation of genes involved in muscle growth (Bennett et al. 2018). During *in planta* infection, the gene transcript is found in the green-yellow module (Table 2) (Lanver et al. 2018). Increased transcription of UMAG_03170 may indicate an increased requirement for ribosomes and may be involved in translating a subset of genes required for later stages of pathogenesis.

UMAG_11281 is the putative ortholog to the protein Prp43 in *S. cerevisiae*. It is predicted to interact with proteins involved in splicing (Suppl. material 2: table S1). One notable interaction is with the *U. maydis* protein UMAG_01091, an rRNA processing protein called SAS10. The *S. cerevisiae* SAS10 ortholog has been shown to have a role in processing the 18S rRNA and has been linked to cell cycle progression (Bernstein and Baserga 2004). During *U. maydis* pathogenesis, the transcript is found in the green module, where the transcript level is low during early pathogenesis, increases, and remains upregulated during late pathogenesis (Table 2) (Lanver et al. 2018). This suggests an increased requirement for this RNA helicase during the later stages of pathogenesis. It may aid in cell cycle progression during *in planta* growth by modulating specific RNPs or facilitating protein-protein interactions during the synthesis of the 18S rRNA.

The *S. cerevisiae* RNA helicase Dbp8 functions during the processing of the 40S ribosomal subunit. The *U. maydis* ortholog, UMAG_10241, is predicted to interact with proteins involved in rRNA processing (Suppl. material 2: table S1). One predicted interaction is with the *U. maydis* ortholog for Esf2, suggesting that UMAG_10241 may function similarly to the *S. cerevisiae* ortholog. Esf2 is an RNA-binding protein capable of enhancing the activity of Dbp8 and guiding it to its binding site (Granneman et al. 2006). The *H. sapiens* ortholog, DDX49, regulates the export of mRNAs and pre-ribosomal RNA levels, which results in the regulation of cell proliferation (Awasthi et al. 2018). The UMAG_10241 transcript is upregulated in the dormant teliospore compared to the haploid and dikaryon cell types and decreases during teliospore germination (Seto et al. 2025). The activity of this RNA helicase may be required to aid in the transition from dormancy to germination by promoting and regulating the translation of genes involved in cellular and metabolic activity.

UMAG_05200 in *U. maydis* is the putative ortholog to the *S. cerevisiae*SF2DEAD-box RNA helicase Dbp10 (Fig. 3, Table 1). STRING analysis predicts that UMAG_05200 interacts with proteins involved in ribosomal biogenesis (Suppl. material 2: table S1), suggesting that its function is conserved across eukaryotes. In *S. cerevisiae*, the helicase activity of Dbp10 is required to induce conformational changes to the pre-rRNA during the maturation of the 60S subunit (Burger et al. 2000; Mitterer et al. 2023). Knockdown and overexpression studies of the ortholog DDX54 in *H. sapiens* cell lines revealed that this RNA helicase responds to DNA damage by binding to pre-mRNAs involved in the DNA damage response pathway to increase their splicing rate and promote cell survival (Milek et al. 2017). The UMAG_05200 transcript is upregulated in the dormant teliospore, and the transcript level is maintained during teliospore germination (Seto et al. 2025). This upregulation during teliospore dormancy may indicate that the transcript is stored and required during germination to respond to potential DNA damage. DNA damage can be caused by genotoxic stressors such as UV exposure and ionizing irradiation, which can reduce fungal spore germination (reviewed in Braga et al. 2015). In the soilborne protozoan *Spongospora subterranea*, there is an enrichment of DNA repair genes during spore germination (Balotf et al. 2021). We hypothesize that UMAG_05200 functions during teliospore germination to protect the growing promycelium by regulating genes that respond to DNA damage.

## ﻿Translation

Messenger RNAs (mRNAs) are exported to the cytoplasm and translated to produce proteins. Translation involves several binding proteins and regulatory elements. The untranslated regions (UTRs) of mRNAs, the 5′ cap, and the poly(A) tail contain control elements that regulate translation. Translation initiation starts with the 40S ribosomal subunit binding near or at the 5′ cap of the mRNA, where the 5′ UTR is scanned for the initiation codon. After the initiation codon is identified, initiation factors are released and the 60S ribosomal subunit is recruited to form the 80S ribosome and begin elongation (reviewed in Wilkie et al. 2003). The translation process involves the function of the following RNA helicases: DDX48, DHX29, and DHX9 in *H. sapiens*, and Tif1/Tif2, Ded1, Dbp5, Dhh1, Hcs1, and Slh1 in *S. cerevisiae*. The *U. maydis* orthologs to these proteins are listed in Table 7. These RNA helicases may function to remodel the mRNA, unwind secondary structures, and recruit proteins to initiate or terminate translation.

**Table 7.** Functions of RNA helicases that are involved in translation. Orthologs in *U. maydis*, *S. cerevisiae*, and *H. sapiens* are listed.

Superfamily	Family	RNA helicase orthologs			RNA helicase function during translation	References
		*U. maydis*	*S. cerevisiae*	*H. sapiens*		
SF1	Upf1-like	UMAG_01122	Hcs1	IGHMBP2	Exact function during translation is unknown; IGHMBP2 is found to bind to the 80S ribosome and tRNA and co-localizes with eIF4G2 and rRNA in the cytoplasm	de Planell-Saguer et al. (2009); Guenther et al. (2009); Jankowsky (2011); Bourgeois et al. (2016)
SF2	DEAD-box	UMAG_06129	Fal1	DDX48	DDX48 may remodel or promote unwinding of seconding structures to allow the 40S to scan for the translation initiation codon	Choe et al. (2014)
		UMAG_05482	eIF4A	eIF4AI eIF4AII	Component of the eIF4F complex that modulates interactions between the 5’ cap and the poly(A) tail eIF4A unwinds secondary structures on the 5’ UTR to allow for ribosome attachment	Li et al. (1999); Svitkin et al. (2001); Shen and Pelletier (2020)
		UMAG_04080	Ded1 Dbp1	DDX3	Uses clamping abilities to pause and/or stabilize the ribosome; Capable of resolving structured 5’ UTRs to initiate translation; Interacts with eIF4G in stress granules to promote and repress translation	Hilliker et al. (2011); Geissler et al. (2012); Sen et al. (2015); Yeter-Alat et al. (2023)
		UMAG_03765	Dbp5	DDX19A DDX19B DDX25	Remodels the mRNA and recruits the protein eRF1 to bind to the stop codon to initiate the process of translation termination	Gross et al. (2007); Mikhailova et al. (2017)
		UMAG_10655	Dhh1	DDX6	Dhh1 may sense a slowing ribosome and is recruited to modulate the mRNA to repress translation. DDX6 may remodel mRNPs by binding to stem-loop structures in the 3’ UTR of the mRNA	Fischer and Weis (2002); Carroll et al. (2011); Sweet et al. (2012); Wang et al. (2015); Shen and Pelletier (2020)
	DEAH/RHA	UMAG_05767		DHX9	May recognize post-transcriptional control elements in the 5’ region of the mRNA and induce conformational changes for efficient translation	Hartman et al. (2006)
		UMAG_00574		DHX29 DHX57	DHX29 is required for efficient translation of mRNAs with highly structured 5’ UTRs by binding to the 40S subunit and inducing conformational changes that allows for the ribosome to position correctly on the mRNA	Pisareva et al. (2008)
	Ski2-like	UMAG_00282	Slh1	HELC1/ASCC3	Slh1 inhibits the translation of mRNAs that lack a poly(A) tail; Induce conformational changes that allow for the ribosome quality control complex to access defective polypeptide chains for degradation	Searfoss and Wickner (2000); Daugeron et al. (2011); Sitron et al. (2017)

Our STRING analysis (Suppl. material 2: table S1) for the *U. maydis* protein UMAG_04665, the putative ortholog to *S. cerevisiae* RNA helicase Dhr1, predicted a protein-protein interaction with the translation elongation factor eEF3 (UMAG_04152). A protein-protein interaction between Dhr1 and eEF3 was reported in *S. cerevisiae* (Sturm et al. 2017); however, whether Dhr1 serves a function during translation has yet to be determined. RNA helicase activity from Dhr1 may be required to remodel the mRNA to bind or displace eEF3 from the mRNA.

Of the RNA helicases that function in translation, we identified five with potential roles in the *U. maydis* life cycle. These RNA helicases are UMAG_04080, UMAG_10655, UMAG_05482, UMAG_00574, and UMAG_01122. The transcripts for UMAG_10655, UMAG_05482, and UMAG_00574 are found in all cell types (Donaldson et al. 2017; Seto et al. 2025). During pathogenesis, these gene transcripts are found in the yellow module (Table 2). UMAG_04080 and UMAG_01122 transcripts are upregulated in the dormant teliospore (Donaldson et al. 2017; Seto et al. 2025) and are found in the magenta and cyan modules, respectively (Table 2). Combining the transcriptome data and current research on their orthologs indicates that these RNA helicases may have roles in *U. maydis* growth, pathogenesis, stress response, and teliospore dormancy and germination.

Extensive research has been conducted on the *S. cerevisiae* RNA helicase Ded1 and its ortholog DDX3 in *H. sapiens*. The *U. maydis* ortholog is identified as UMAG_04080, and its function in RNA metabolism is predicted to be conserved based on our STRING analysis (Suppl. material 2: table S1). UMAG_04080 was identified as an RNA helicase with gene transcript levels upregulated in the dormant teliospore, decreased once germination was initiated, and remained relatively unchanged during the remainder of the germination time course (Seto et al. 2025). This transcript pattern suggests that UMAG_04080 may have a function during the exit from teliospore dormancy to germination. Deletion mutants in *U. maydis* are nonviable; however, mutants were created in which UMAG_04080 was expressed in an ectopic location under a carbon-sensitive promoter, and UMAG_04080 was deleted from its native locus. We subsequently named this RNA helicase *uded1* in *U. maydis* (Seto and Saville 2025). Characterization of these mutants showed that when grown on solid medium, slow growth and mycelial growth phenotypes were observed. Normal budding haploid growth was restored when the solid medium was supplemented with the sugar alcohol sorbitol (Seto and Saville 2025). Sorbitol is an osmoprotectant that is often added to medium for cultivating fungi with fragile cell walls (Górka-Nieć et al. 2010). Overexpression of *DED1* in *S. cerevisiae* can cause growth defects and can drive stress granule formation by binding to mRNAs to repress translation and sequester these mRNAs in the stress granules (Aryanpur et al. 2017; Aryanpur et al. 2022). In *S. pombe*, overexpression of *ded1* interferes with the response of the MAPK pathway by negatively regulating the cell cycle response to osmotic stressors (Forbes et al. 1998). Upregulation of *uded1* impacts *U. maydis* growth and may impact the MAPK pathway, contributing to the observed slow growth and mycelial phenotypes. The restoration of budding growth in the presence of sorbitol suggests that altered expression of *uded1* may result in a defect in cell wall formation, making cells more sensitive to osmotic stress, or may impair mitotic division (Seto and Saville 2025). We hypothesize that *uded1* functions to repress translation during teliospore dormancy by binding to mRNAs to create an mRNP that is stabilized. The mRNP is stored and disassembled when germination is initiated, making the mRNAs available for translation (Seto and Saville 2025).

UMAG_10655 was identified as the putative *U. maydis* ortholog to Dhh1 in *S. cerevisiae* (Feldbrügge et al. 2008). Our STRING analysis (Suppl. material 2: table S1) predicts that UMAG_10655 interacts with proteins involved in P-body and stress granule assembly, suggesting that the *U. maydis* ortholog may function similarly to Dhh1 in other organisms. The *C. neoformans* ortholog, Vad1, was found to regulate stress response and the expression of a subset of virulence genes (Panepinto et al. 2005). It was identified as a central regulator of virulence genes and promotes resistance to the host immune response (Qiu et al. 2013). During pathogenesis, the transcript is found in the yellow module (Table 2) and is co-expressed with genes involved in metabolism and cellular activity (Lanver et al. 2018). The function of UMAG_10655 is predicted to be similar to its orthologs in that it may respond to environmental stressors or changes in the host plant defense system to regulate translation of a subset of genes, enhancing successful penetration of the plant to cause disease.

The putative eIF4A ortholog in *U. maydis* is UMAG_05482 (Fig. 3, Table 1), and the STRING analysis (Suppl. material 2: table S1) suggests a conserved function in translation initiation. eIF4A is a subunit of the eIF4F complex, which is a component of the translational machinery. Heat shock disassembles the eIF4F complex, which causes translation repression of mRNAs such as housekeeping genes. eIF4G and eIF4E assemble to form mRNPs sequestered in heat stress granules. eIF4A is unaffected by heat shock stress and functions independently to promote the translation of mRNAs that respond to heat stress (Desroches Altamirano et al. 2024). We hypothesize that altering the expression of UMAG_05482 may impact the heat stress response of *U. maydis*. During pathogenesis, the transcript is found in the yellow module (Table 2). In this module, there is an increase in gene transcripts that pertain to protein catabolism and autophagy (Lanver et al. 2018). This transcript profile suggests that the enhancement of UMAG_05482 during the early stages of pathogenesis is likely to facilitate the translation of specific genes required for this stage of pathogenesis.

UMAG_00574 was identified as the putative ortholog to the *H. sapiens* RNA helicases DHX29 and DHX57. An ortholog in the ascomycete fungi was not found; however, orthologs were found in the basidiomycetes (Fig. 4). The STRING analysis for UMAG_00574 predicted protein-protein interactions with ribosomal and translation initiation proteins (Suppl. material 2: table S1). Three notable protein-protein interactions predicted by STRING are with the proteins UMAG_03144, UMAG_10988, and UMAG_04249. The KEGG pathway for these proteins indicates that they are involved in endocytosis. This suggests that RNA helicase activity may be involved in assembling the endocytic machinery. DHX29 binds to the 40S ribosomal subunit to induce conformational changes, which allows the ribosome to position itself correctly on mRNAs containing highly structured 5′ UTRs (Pisareva et al. 2008). Messenger RNAs with highly structured 5′ UTRs are typically highly regulated and require multiple layers of gene expression control (Leppek et al. 2018). UMAG_00574 is found in the yellow module during pathogenesis (Table 2) and may function to efficiently regulate the translation of regulatory genes, such as metabolic and cellular activity genes also found in this module. For example, the endocytic pathway contributes to the early stages of *U. maydis* pathogenesis (Fuchs and Steinberg 2005; Fuchs et al. 2006), and UMAG_00574 may contribute to regulating the translation of genes involved in this pathway.

Protein sequence and phylogenetic analysis identified UMAG_01122 as the ortholog to *S. cerevisiae* Hcs1 and *H. sapiens* IGHMBP2 (Table 1, Fig. 2). It is a member of the SF1 superfamily of Upf1-like RNA helicases; however, there are limited studies on the function of this RNA helicase. Currently, Hcs1/IGHMBP2 has been demonstrated to interact with the translational machinery and is suggested to function during translation, but its exact role remains unclear (de Planell-Saguer et al. 2009; Guenther et al. 2009; Jankowsky 2011; Bourgeois et al. 2016). Our STRING analysis predicts UMAG_01122 interacts with UMAG_10602, the putative ortholog to Sen1/SETX (Suppl. material 2: table S1), suggesting an additional role in transcription. Transcriptome analysis during *U. maydis* pathogenesis indicates that the transcript is in the cyan/tumor development module (Table 2) (Lanver et al. 2018) and is upregulated in the dormant teliospore (Donaldson et al. 2017; Seto et al. 2025). If UMAG_01122 functions during translation, it may aid in translating genes required for tumor and teliospore development, with its transcript stored in the dormant teliospore. During teliospore germination, UMAG_01122 could interact with UMAG_10602 to regulate gene expression of a subset of genes or aid in maintaining genome stability and preventing R-loop formation during transcription.

## ﻿RNA degradation

Degradation of RNA is a highly efficient and conserved process that involves numerous enzymes, many of which target the same RNA molecules. This process removes byproducts generated during RNA processing, such as excised introns, regulates gene expression through the removal of mRNAs, and serves as a quality control mechanism to eliminate defective RNAs and RNPs (Parker 2012). There are three enzyme classes capable of RNA degradation: endonucleases, 5′ exonucleases, and 3′ exonucleases. RNA degradation also depends on the action of helicases, polymerases, and chaperones (Houseley and Tollervey 2009). RNA helicases that function during RNA degradation include Dhh1, Ski2, Upf1, Dbp2, and Mtr4 in *S. cerevisiae*. Their *U. maydis* orthologs and specific functions are summarized in Table 8.

**Table 8.** RNA helicases involved in RNA degradation. Orthologs in *U. maydis*, *S. cerevisiae*, and *H. sapiens* are listed.

Superfamily	Family	RNA helicase orthologs			RNA helicase function during RNA degradation	References
		*U. maydis*	*S. cerevisiae*	*H. sapiens*		
SF1	Upf1-like	UMAG_11428	Upf1	UPF1	Is recruited to mRNAs that contain a long 3’ UTR and interacts with NCBP1 to begin the initial steps of the NMD pathway	Hogg and Goff (2010); Imamachi et al. (2012); Ganesan et al. (2022)
SF2	DEAD-box	UMAG_10655	Dhh1	DDX6	Dhh1 activates decapping of the 5’ end by modulating the structure of the mRNA to facilitate access for Dcp1	Fischer and Weis (2002); Sweet et al. (2012)
		UMAG_10095	Dbp2	DDX5 DDX17	May act as an RNA binding protein to facilitate or stabilize the assembly of the NMD machinery on the mRNA	Xing et al. (2019)
	Ski2-like	UMAG_00393	Ski2	SkiV2	Ski2 unwinds secondary structures and resolve RNPs at the 3’ end of the mRNA for exosome access	Anderson and Parker (1998); Johnson and Jackson (2013)
		UMAG_11667	Mtr4	MTREX	Mtr4 may target aberrant precursor 5.8S rRNA for degradation; May function to unwind RNA to generate long stretches of sRNA to allow the exosome to access	de la Cruz et al. (1998b); Jia et al. (2011)

RNA decay for many mRNAs is initiated by the shortening of the poly(A) tail, called deadenylation. This is not a uniform process, and the transition is reflective of changes in mRNP composition or RNA structure. Once the poly(A) tail is reduced to five to fifteen adenosines, degradation is triggered (Decker and Parker 1994). RNA degradation occurs via one of two major pathways. The most common pathway involves removal of the 5’ cap by the decapping enzyme Dcp1/Dcp2, which initiates degradation in the 5′-to-3′ direction by the enzyme Xrn1. This process may involve the function of the RNA helicase Dhh1 in *S. cerevisiae* and its *U. maydis* ortholog UMAG_10655 (Fischer and Weis 2002; Sweet et al. 2012). Degradation in the 3′-to-5′ direction is the second pathway and involves the exosome and other cofactors. The exosome requires interaction with several Ski proteins, one of which is the RNA helicase Ski2 in *S. cerevisiae* (Anderson and Parker 1998; Parker 2012; Johnson and Jackson 2013). The *U. maydis* Ski2 ortholog was previously identified as UMAG_00393 (Feldbrügge et al. 2008).

The quality control pathways ensure that defective RNAs are identified and directed to degradation. The nonsense-mediated decay (NMD) pathway is one surveillance mechanism responsible for degrading mRNAs with aberrant translation termination. NMD pathway activation is facilitated through the function of RNA helicase Upf1. The *U. maydis* ortholog was previously identified as UMAG_11428 (Feldbrügge et al. 2008; Martinez-Montiel et al. 2016). Once activated, the NMD machinery is assembled and stabilized on the mRNA with the function of RNA helicase Dbp2 (Xing et al. 2019). The mRNA is subjected to rapid deadenylation and increased rates of 3′-to-5′ degradation (Parker 2012). Another quality control pathway is the no-go decay (NGD) pathway, which degrades mRNAs that stall during translation due to strong stem loops, rare codons, or sites of depurination. Targets of the NGD pathway are subjected to endonucleolytic cleavage, followed by degradation of the 3′ mRNA fragment by Xrn1 and the 5′ mRNA fragment by the exosome (Parker 2012). The last quality control mechanism is the nonstop decay (NSD) pathway, which targets mRNAs lacking a translation termination codon. It is triggered when the ribosome reaches the 3′ end of the mRNA and translation termination cannot be achieved (Parker 2012).

Our transcriptome analysis identified UMAG_00393 as an RNA helicase with potential roles in *U. maydis* pathogenesis and stress response. The transcript is present in all cell types and during teliospore germination (Donaldson et al. 2017; Seto et al. 2025) and is found in the yellow module during pathogenesis (Table 2) (Lanver et al. 2018). Our analysis identified it as the ortholog to Ski2 (Fig. 5, Table 1). UMAG_00393 is predicted to function in RNA degradation based on the STRING results (Suppl. material 2: table S1). In *C. neoformans*, Ski2 deletion mutants were sensitive to high temperatures due to defects in cell wall integrity. The cell walls of these mutants showed abnormal chitin distribution, suggesting that Ski2 may play a role in chitin production. Additionally, these mutants exhibited decreased virulence and increased sensitivity to osmotic stress and drugs that inhibit ribosomal function (Li et al. 2022). We hypothesize that UMAG_00393 deletion mutants will show an attenuated response to osmotic stress and cell wall defects. This transcript profile suggests an increased requirement for UMAG_00393 during the early stages of pathogenesis, likely to support the increased transcription and translation of RNAs for cellular growth and metabolism within the plant.

## ﻿Mitochondrial RNA processing

The mitochondrial genome requires the function of several factors for maintenance and expression. Many proteins essential to mitochondrial function are encoded by nuclear genes and imported into the mitochondria. The mitochondrial genome itself also encodes proteins that are critical for mitochondrial activity. The expression and regulation of nuclear and mitochondrial genes are coordinated by an active RNA processing system that balances the rates of RNA synthesis and degradation (Nicholls et al. 2013; Markov et al. 2014). The processing and maturation of mitochondrial mRNA in humans is more straightforward than in *S. cerevisiae*. Human mitochondrial mRNA does not require splicing machinery, as the genes do not contain introns. The transcripts are polyadenylated by the mitochondrial poly(A) polymerase and are not capped at the 5′ end (Nicholls et al. 2013). In *S. cerevisiae*, transcribed mitochondrial DNA contains long 3′ UTRs and is not polyadenylated. Transcripts can contain introns, and splicing machinery is required to excise them. The machinery responsible for the transcription and splicing of these transcripts includes the function of RNA helicase *Mss116* (Niemer et al. 1995; Minczuk et al. 2002; Markov et al. 2014). Mitochondria also possess a degradation system that requires the RNA helicase *Suv3* in *S. cerevisiae* (Dziembowski et al. 2003; Guo et al. 2011; Szczesny et al. 2013). The *U. maydis* mitochondrial genome has genes that contain introns (Pfeifer et al. 2012) and may require the function of RNA helicases such as *Mss116* and *Suv3* during RNA processing. Table 9 indicates the *U. maydis* orthologs and outlines the specific functions of the *S. cerevisiae* RNA helicases in the mitochondria.

**Table 9.** RNA helicases that are involved in mitochondrial RNA processing. Orthologs for *U. maydis*, *S. cerevisiae*, and *H. sapiens* are listed.

Superfamily	Family	RNA helicase orthologs			RNA helicase function during mitochondrial RNA processing	References
		*U. maydis*	*S. cerevisiae*	*H. sapiens*		
SF2	DEAD-box	UMAG_00652 UMAG_06314	Mss116		Modulates mitochondrial transcription and is involved in the splicing of group I and II mitochondrial introns	Niemer et al. (1995); Minczuk et al. (2002); Markov et al. (2014)
	Ski2-like	UMAG_04997	Suv3	SUV3	Is involved in the processing of the r1 intron in the precursor of the mitochondrial 21S rRNA; Functions within the mitochondrial degradosome to unwind dsRNA	Stepien et al. (1995); Dziembowski et al. (2003); Guo et al. (2011)

## ﻿RNA helicases with unknown functions

**Figure 6.**
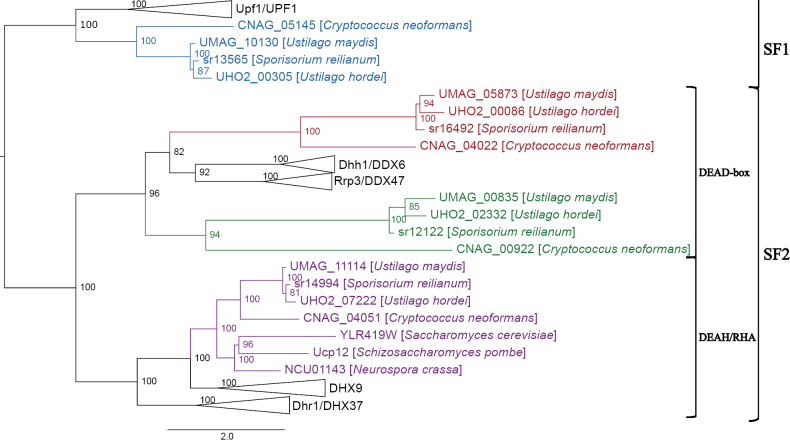
Maximum likelihood phylogenetic tree of RNA helicases with unknown function. The tree was constructed using orthologs of RNA helicases with unknown functions from *U. maydis*, *S. reilianum*, *U. hordei*, *C. neoformans*, *S. cerevisiae*, *S. pombe*, *N. crassa*, *A. thaliana*, *C. elegans*, *D. discoideum*, and *H. sapiens*, using W-IQ-TREE multicore version 1.6.12 with default settings (Trifinopoulos et al. 2016), 1000 ultrafast bootstrap replicates, and the approximate Bayes test. The tree was visualized with FigTree v1.4.4 (http://tree.bio.ed.ac.uk/software/figtree/), rooted with the SF1 clade as the outgroup. Bootstrap values are indicated at each node. Phylogenetic groups containing known RNA helicases were collapsed and labeled with their *S. cerevisiae* and *H. sapiens* orthologs. Clades highlighted in blue, red, green, and purple indicate RNA helicases with unknown functions. The scale bar represents the expected number of substitutions per amino acid. The full phylogenetic tree, including all gene names and species, is provided in Suppl. material 1: fig. S5.

Our sequence and phylogenetic analyses identified several RNA helicases that are either fungal-specific or whose function is unknown. We identified the SF1 Upf1-like RNA helicase UMAG_10130 (Figs 1, 6) and the SF2DEAD-box RNA helicases UMAG_00835 and UMAG_05873 (Figs 3, 6) as putative basidiomycete-specific RNA helicases. The SF2 DEAH RNA helicase YLF419W in *S. cerevisiae* has orthologs only in the fungal species we assessed, and the *U. maydis* putative ortholog is UMAG_11114 (Figs 4, 6).

The *U. maydis* gene UMAG_10130 is an SF1 Upf1-like RNA helicase, and the phylogenetic analysis shows a separate clade containing genes in the other basidiomycete fungi, *S. reilianum*, *U. hordei*, and *C. neoformans*, but not with any other species we assessed (Figs 2, 6). Sequence analysis indicates that UMAG_10130 contains sequence motifs typical of RNA helicases in the SF1 Upf1-like family, indicating that a helicase core is present. UMAG_10130 and its basidiomycete orthologs have not been characterized within their respective organisms. The STRING analysis (Suppl. material 2: table S1) predicts putative protein-protein interactions with the same proteins as UMAG_11428, the ortholog to Upf1. Upf1 is an RNA helicase that functions during RNA degradation, where it activates the NMD pathway. UMAG_10130 may function similarly to UMAG_11428 in the NMD pathway. The gene transcript is found in all *U. maydis* cell types (Donaldson et al. 2017; Seto et al. 2025) and is found in the magenta module during pathogenesis (Table 2). The magenta module contains genes that have a significant role during the early stages of pathogenesis, more specifically during the establishment and maintenance of biotrophy (Lanver et al. 2018). The UMAG_10130 transcript is found in a different module from UMAG_11428, suggesting that it may assist in the degradation of a specific subset of genes during this stage of pathogenesis that is different from those targeted by UMAG_11428.

The phylogenetic analysis identified two SF2DEAD-box RNA helicases with no other closely related orthologs other than those found in the *Basidiomycota*. These RNA helicases have been identified as UMAG_05873 and UMAG_00835 (Figs 3, 6) and are uncharacterized proteins in *U. maydis*. Our findings suggest that these RNA helicases are basidiomycete-specific RNA helicases; however, more fungal species would need to be assessed for this interpretation to be conclusive. Sequence analysis revealed that both genes have a helicase core containing several canonical RNA helicase sequence motifs typical of this RNA helicase family (data not shown). The STRING analysis predicts that UMAG_05873 interacts with translational pathway proteins (Suppl. material 2: table S1), suggesting a role in translation. Analysis of the UMAG_05873 transcript during in planta infection places the transcript in the magenta module (Lanver et al. 2018). This module is correlated to biotrophic establishment and maintenance. The enhancement of this RNA helicase during this stage of pathogenesis suggests it may function to aid in the translation of transcripts required for changes in the growth of the mycelium during pathogenesis that may be specific to basidiomycetes. STRING analysis for UMAG_00835 predicts protein-protein interactions with ribosome biogenesis proteins, suggesting a role in processing and assembling ribosomes (Suppl. material 2: table S1). The UMAG_00835 transcript is present in the cyan/tumor module during in planta growth, which contains genes that respond to nutrient limitations on the plant surface (Table 2). The transcript is upregulated in the dormant teliospore and is decreased during germination (Seto et al. 2025). This transcript pattern suggests that UMAG_00835 is stored and is immediately translated during germination. Its presence in the Lanver et al. (2018) cyan/tumor module suggests that this RNA helicase may respond to changes in external nutrients. We hypothesize that when dormant teliospores are in a nutrient-rich environment, a decrease in this RNA helicase during germination promotes the translation of genes for cellular growth.

An RHA-group SF2 DEAH RNA helicase YLF419W in *S. cerevisiae* was identified by Jankowsky (2011). Our analysis identified UMAG_11114 as the *U. maydis* ortholog. This RNA helicase is largely uncharacterized. Based on current research, it is a nonessential gene (Shiratori et al. 1999; Colley et al. 2000), and the protein has been found in both the mitochondria (Sickmann et al. 2003) and cytoplasm (Huh et al. 2003). YLF419W has protein sequence similarities to ancient eukaryotic retinoblastoma (Rb) protein, suggesting that YLF419W evolved from an ancient Rb gene, lost its Rb function, and instead gained the function as an RNA helicase (Takemura 2005). Our phylogenetic analysis (Figs 4, 6) shows that YLF419W clusters with putative RNA helicases in other fungal species.

UMAG_11114 was identified as an RNA helicase that currently has an unknown function. The *N. crassa* ortholog was identified as *msp-8* (NCU01143), and its putative function is in pre-mRNA splicing (Adhvaryu et al. 2016). Our STRING analysis predicted protein-protein interactions with the same proteins as UMAG_00574, suggesting that this RNA helicase may have a redundant function. During *U. maydis* pathogenesis, the UMAG_11114 transcript is in the yellow module (Table 2). This transcript pattern indicates upregulation of the transcript during the mid to later stages of pathogenesis. This suggests a role in supporting post-transcriptional regulation of highly metabolic and cellular activity genes (Lanver et al. 2018). Further functional characterization in *U. maydis* is required to determine the role this RNA helicase has in RNA metabolism and if there is an impact on the progression of pathogenesis.

## ﻿Conclusion

Annotating the RNA helicases of the basidiomycete *U. maydis* allowed the identification of their functions in relation to gene regulation and response to environmental factors. This provided insights into their potential roles during fungal growth and development. For pathogenic fungi, RNA helicases have the potential to modulate disease progression within their host. Our analysis identified 46 RNA helicases within *U. maydis*. Through a comprehensive review of the current research on their orthologs, we were able to make predictions of their functions and roles in the life cycle. We identified 28 RNA helicases that may contribute to *U. maydis* growth, stress response, pathogenesis, or teliospore dormancy and germination. Further characterization of these RNA helicases and their influence on the various aspects of the fungal life cycle and pathogenicity can aid in developing methods for mitigating or preventing fungal diseases.

## ﻿Additional information

### Use of AI

No use of AI was reported.

### Adherence to national and international regulations

All the fungal strains used in this study have been legally obtained, respecting the Convention on Biological Diversity (Rio Convention).

### Author ORCIDs

Amanda M. Seto https://orcid.org/0009-0006-1838-7014

Barry J. Saville https://orcid.org/0000-0003-2949-7156

### Data availability

All of the data that support the findings of this study are available in the main text or Supplementary Information.

## Supplementary materials

Supplementary material 1 Supplementary figures Data type docx Explanation note **figure S1**: The original maximum likelihood phylogenetic tree of SF1 Upf1-like RNA helicases; **figure S2**: The original maximum likelihood phylogenetic tree of SF2 DEADbox RNA helicases; **figure S3**: The original maximum likelihood phylogenetic tree of SF2 DEAH RNA helicases; **figure S4**: The original maximum likelihood phylogenetic tree of SF2 Ski2-lie RNA helicases. https://binary.pensoft.net/file/1428929This dataset is made available under the Open Database License (http://opendatacommons.org/licenses/odbl/1.0/). The Open Database License (ODbL) is a license agreement intended to allow users to freely share, modify, and use this Dataset while maintaining this same freedom for others, provided that the original source and author(s) are credited.Amanda M. Seto, Barry J. Saville

Supplementary material 2 Supplementary tables Data type xlsx Explanation note **table S1**: STRING v. 12.0 – Predicted Functional Partners. https://binary.pensoft.net/file/1428930This dataset is made available under the Open Database License (http://opendatacommons.org/licenses/odbl/1.0/). The Open Database License (ODbL) is a license agreement intended to allow users to freely share, modify, and use this Dataset while maintaining this same freedom for others, provided that the original source and author(s) are credited.Amanda M. Seto, Barry J. Saville
