# Supplementary material for: ﻿Annotation and functional prediction of RNA helicases in Ustilago maydis
Source: IMA Fungus. 2025 Oct 2;16:e151785. doi: 10.3897/imafungus.16.151785 (PMC12511879; doi:10.3897/imafungus.16.151785)
Supplement: Supplementary material 1 — Supplementary figures [file imafungus-16-e151785-s001.docx]

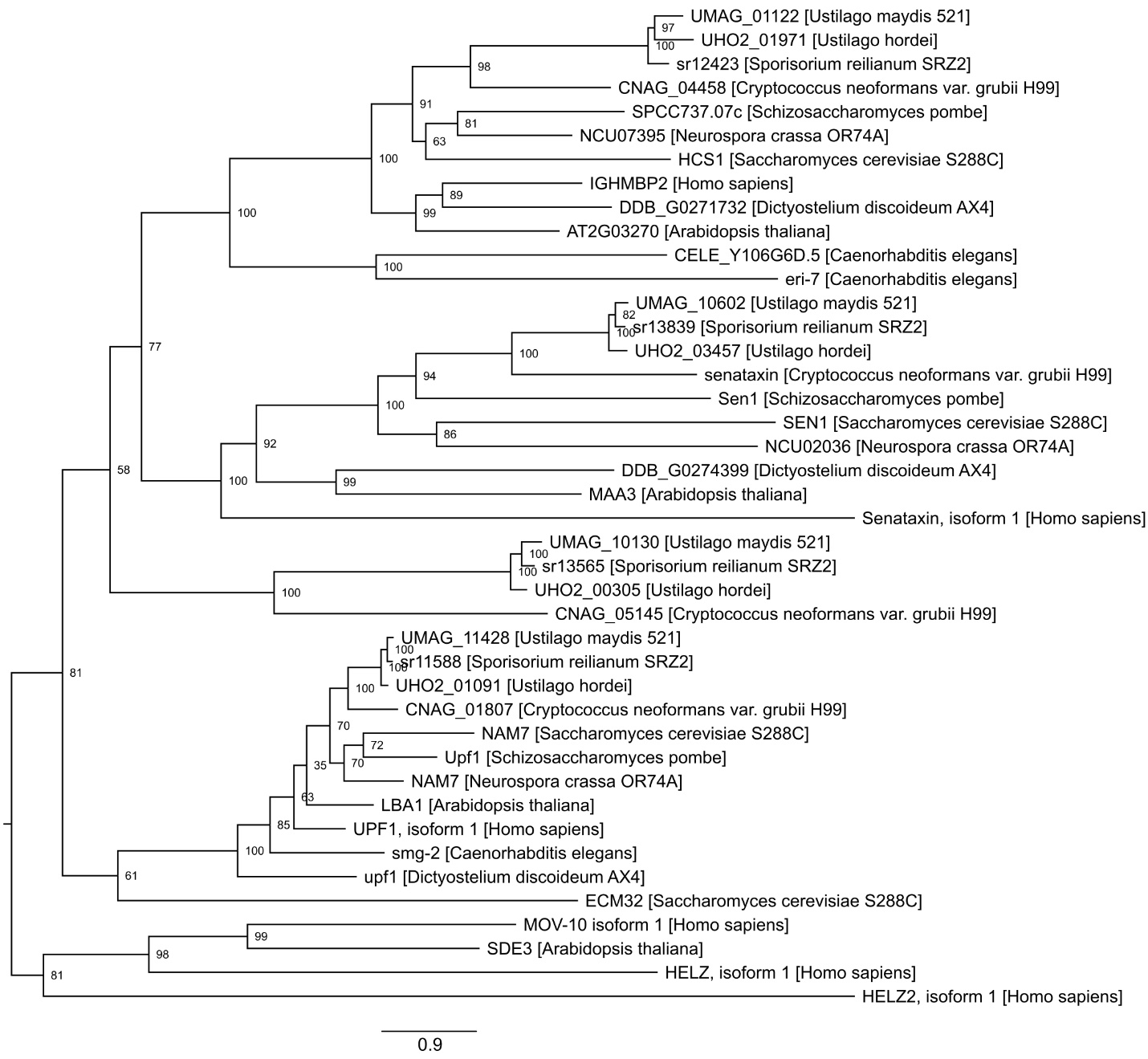


**Figure S1.** The original maximum likelihood phylogenetic tree of SF1 Upf1-like RNA helicases. The phylogenetic tree was created with orthologs from *U. maydis*, *S. reilianum*, *U. hordei*, *C. neoformans*, *S. cerevisiae*, *S. pombe*, *N. crassa*, *A. thaliana*, *C. elegans*, *D. discoideum*, and *H. sapiens* using W-IQ-Tree multicore version 1.6.12 with default settings (Trifinopoulos et al. 2016), 1000 ultrafast bootstrap alignments, and approximate Bayes test. The tree was visualized with FigTree v1.4.4 (<http://tree.bio.ed.ac.uk/software/figtree/>), rooted at the midpoint, and the bootstrap value is indicated for each node. The scale bar indicates the expected number of substitutions per amino acid.


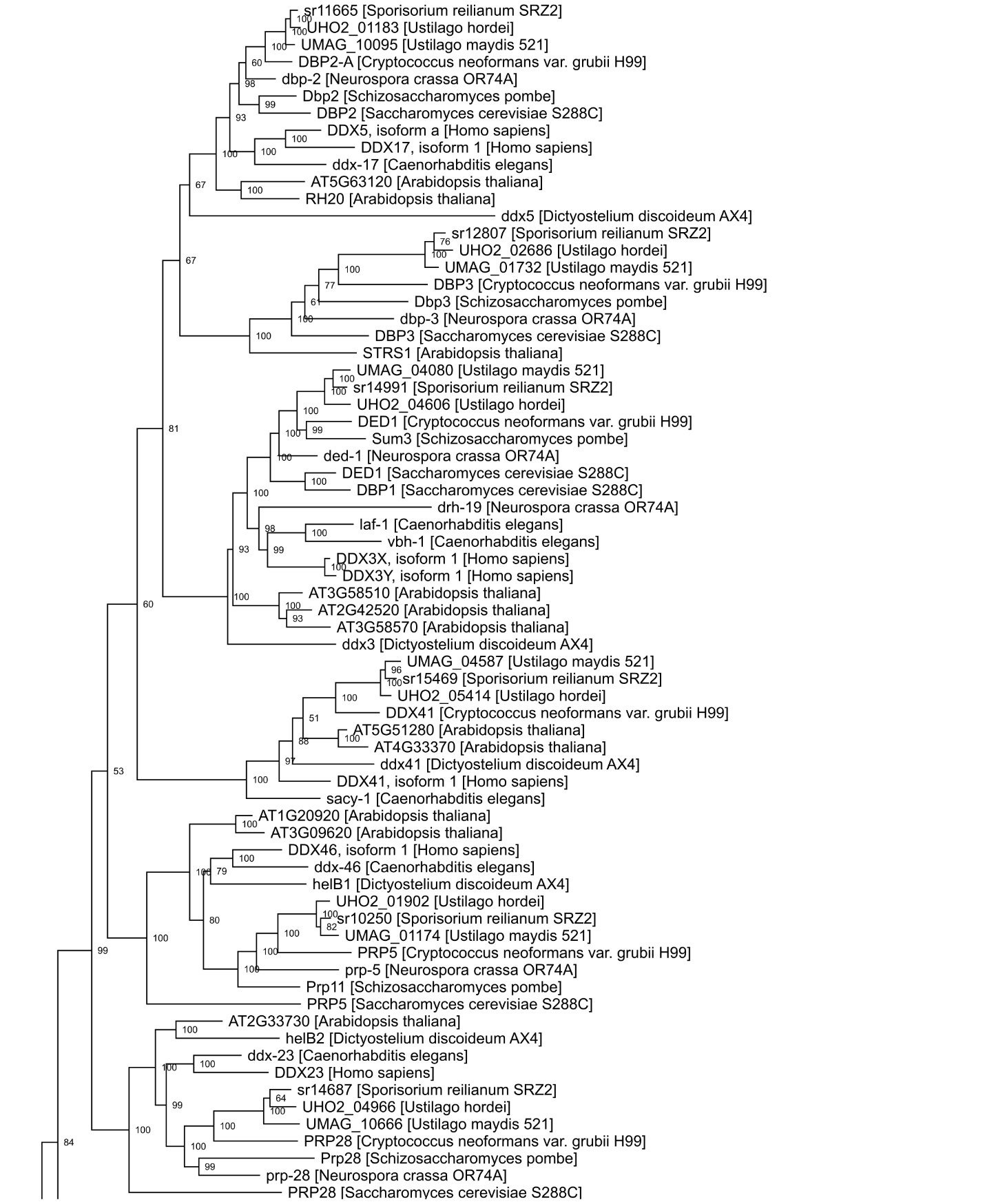

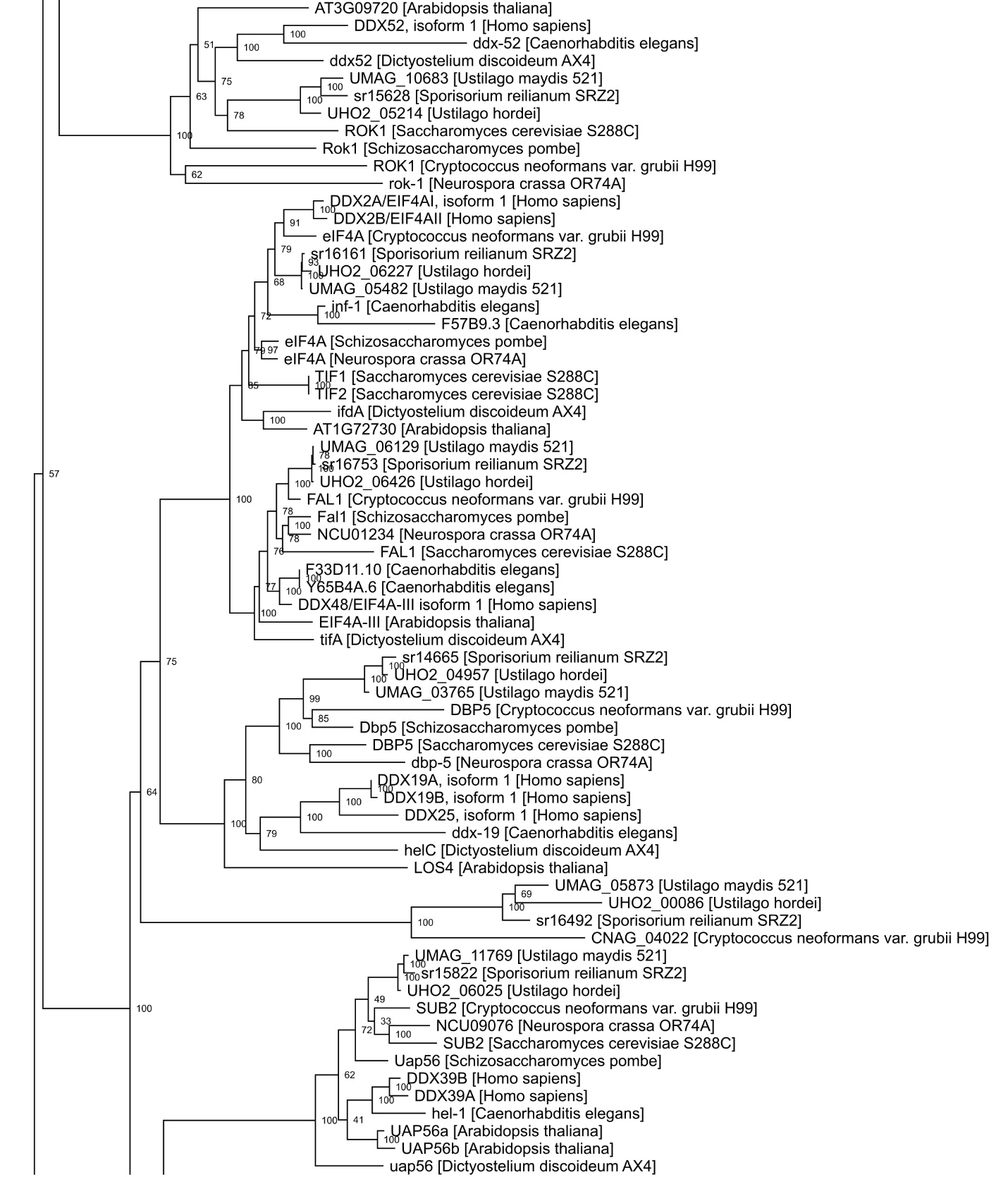

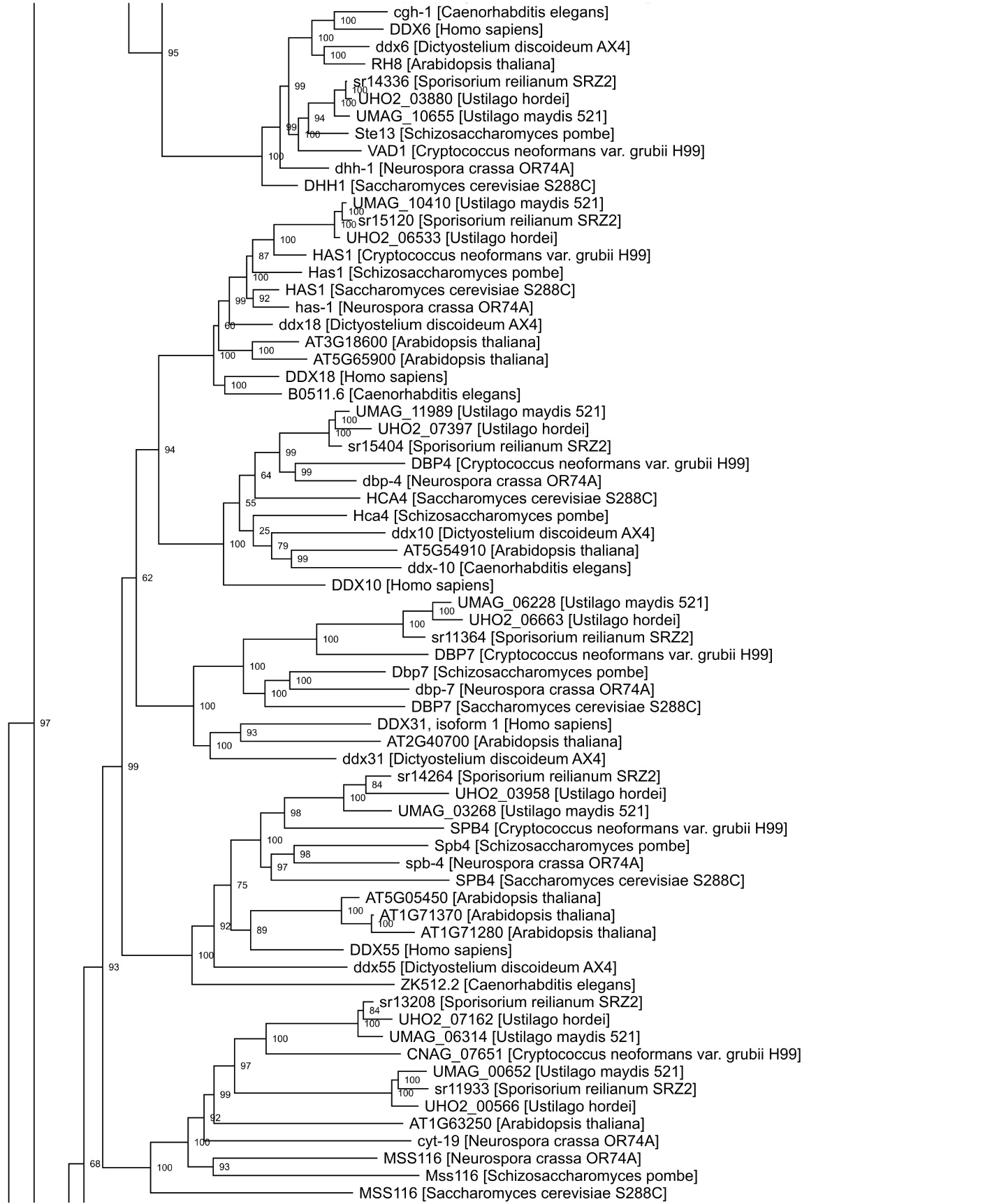

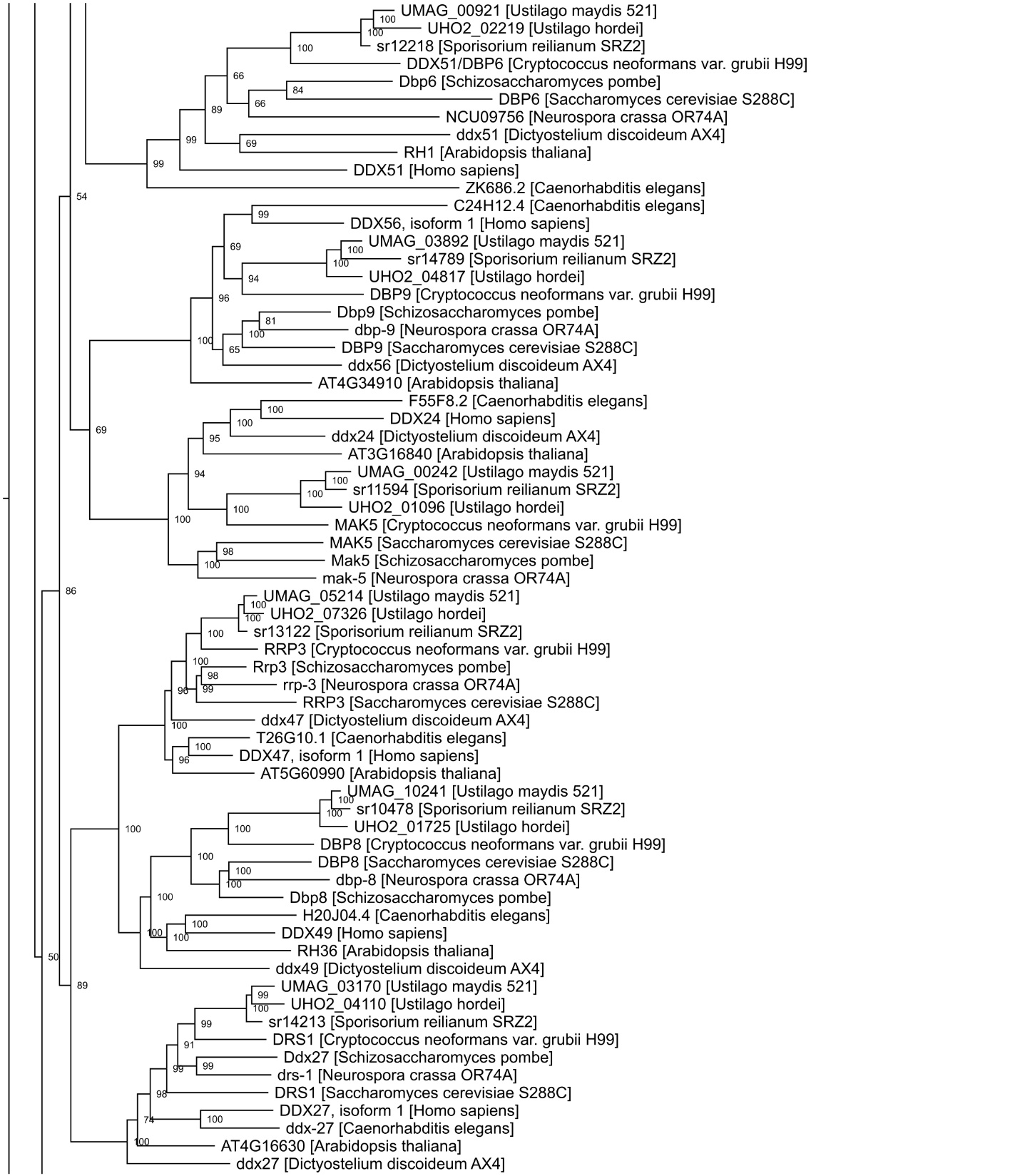

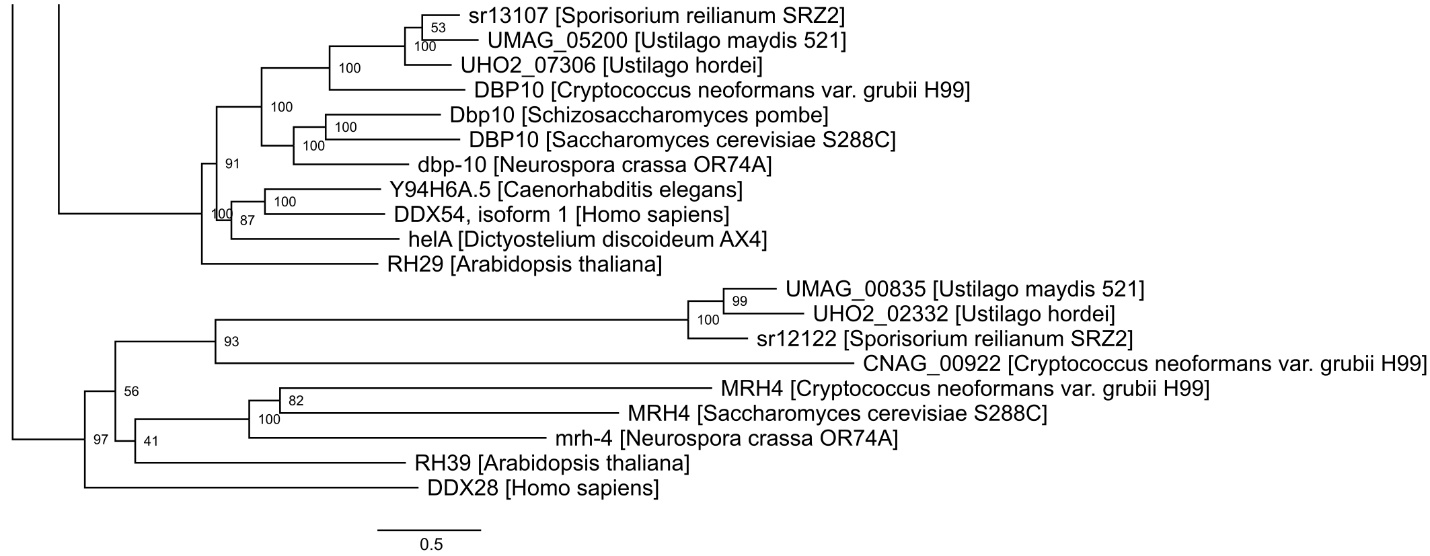
**Figure S2.** The original maximum likelihood phylogenetic tree of SF2 DEADbox RNA helicases. The phylogenetic tree was created with orthologs from *U. maydis*, *S. reilianum*, *U. hordei*, *C. neoformans*, *S. cerevisiae*, *S. pombe*, *N. crassa*, *A. thaliana*, *C. elegans*, *D. discoideum*, and *H. sapiens* using W-IQ-Tree multicore version 1.6.12 with default settings (Trifinopoulos et al. 2016), 1000 ultrafast bootstrap alignments, and approximate Bayes test. The tree was visualized with FigTree v1.4.4 (<http://tree.bio.ed.ac.uk/software/figtree/>), rooted at the midpoint, and the bootstrap value is indicated for each node. The scale bar indicates the expected number of substitutions per amino acid.


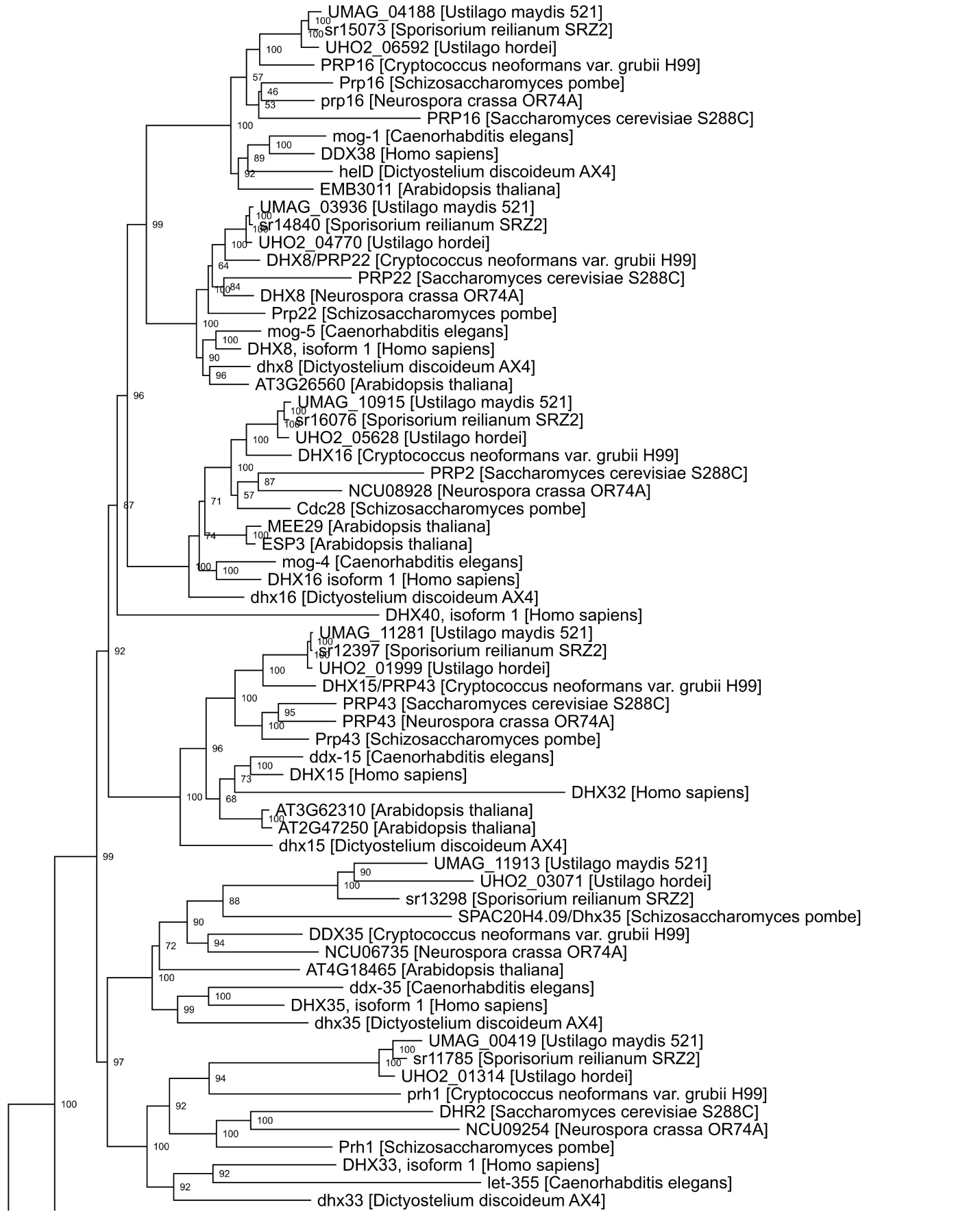

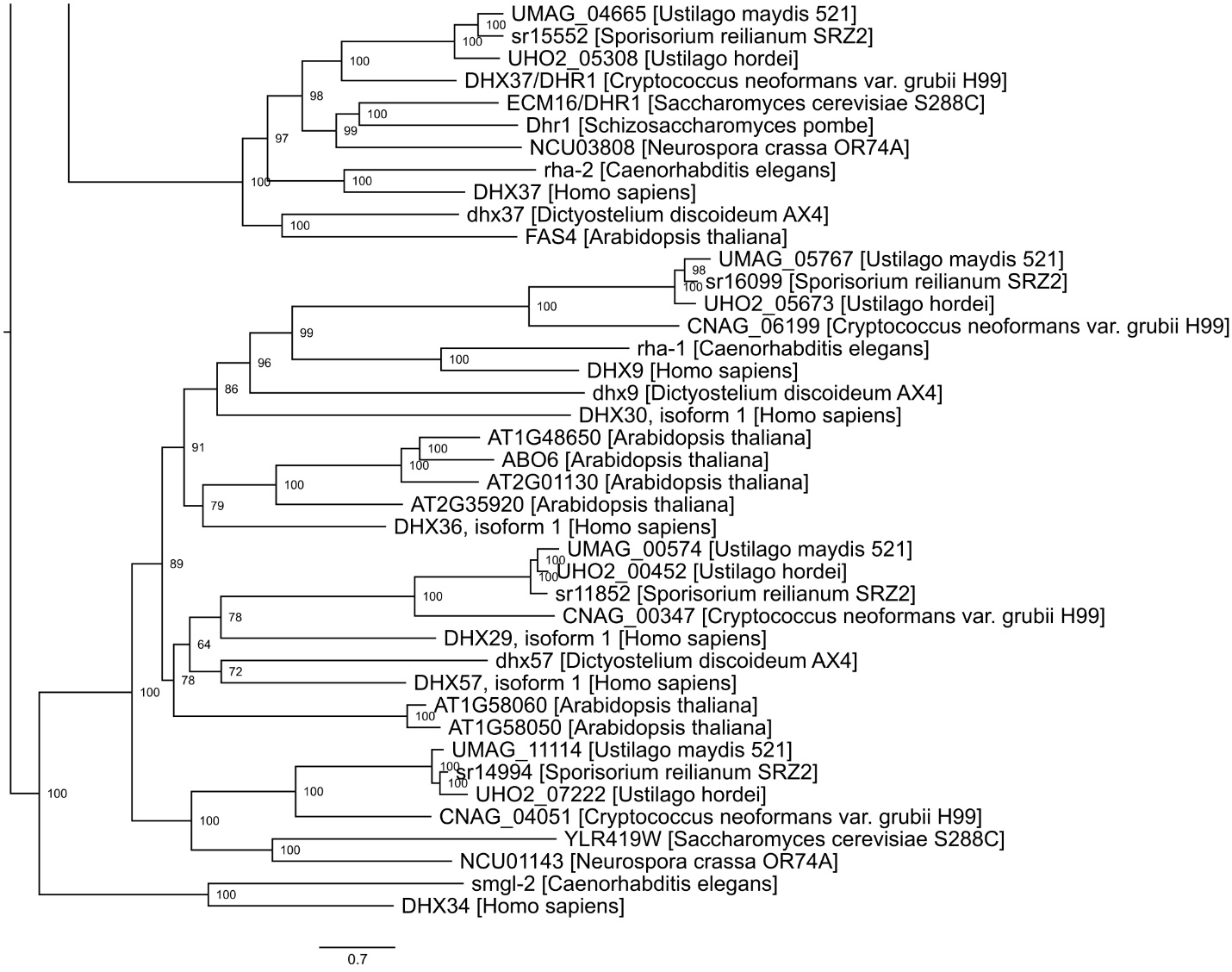
**Figure S3.** The original maximum likelihood phylogenetic tree of SF2 DEAH RNA helicases. The phylogenetic tree was created with orthologs from *U. maydis*, *S. reilianum*, *U. hordei*, *C. neoformans*, *S. cerevisiae*, *S. pombe*, *N. crassa*, *A. thaliana*, *C. elegans*, *D. discoideum*, and *H. sapiens* using W-IQ-Tree multicore version 1.6.12 with default settings (Trifinopoulos et al. 2016), 1000 ultrafast bootstrap alignments, and approximate Bayes test. The tree was visualized with FigTree v1.4.4 (<http://tree.bio.ed.ac.uk/software/figtree/>), rooted at the midpoint, and the bootstrap value is indicated for each node. The scale bar indicates the expected number of substitutions per amino acid.


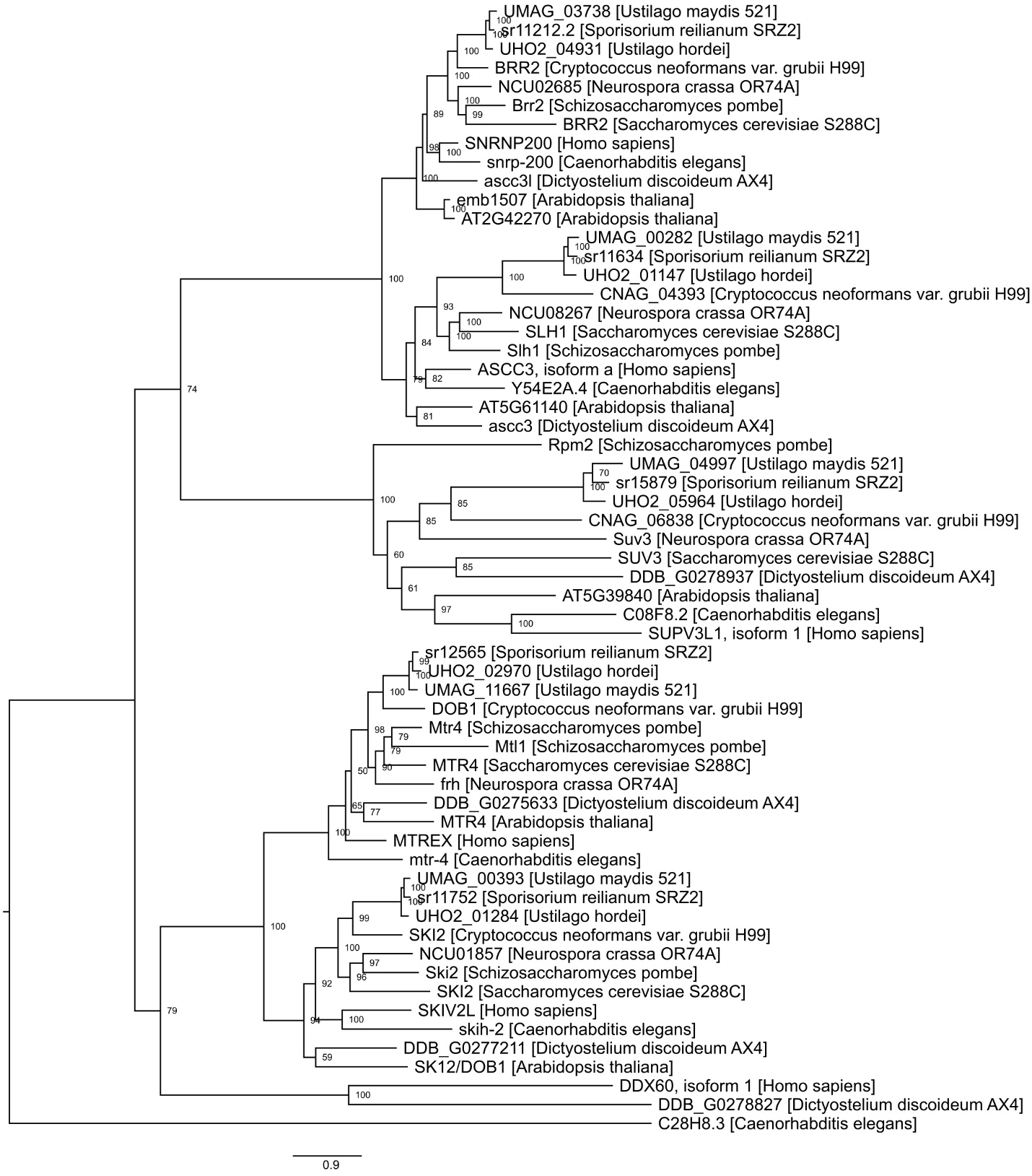
**Figure S4.** The original maximum likelihood phylogenetic tree of SF2 Ski2-lie RNA helicases. The phylogenetic tree was created with orthologs from *U. maydis*, *S. reilianum*, *U. hordei*, *C. neoformans*, *S. cerevisiae*, *S. pombe*, *N. crassa*, *A. thaliana*, *C. elegans*, *D. discoideum*, and *H. sapiens* using W-IQ-Tree multicore version 1.6.12 with default settings (Trifinopoulos et al. 2016), 1000 ultrafast bootstrap alignments, and approximate Bayes test. The tree was visualized with FigTree v1.4.4 (<http://tree.bio.ed.ac.uk/software/figtree/>), rooted at the midpoint, and the bootstrap value is indicated for each node. The scale bar indicates the expected number of substitutions per amino acid.

**References**

Trifinopoulos J, Nguyen L-T, von Haeseler A, Minh BQ (2016) W-IQ-TREE: a fast online phylogenetic tool for maximum likelihood analysis. Nucleic Acids Research 44: W232-W235. doi:10.1093/nar/gkw256.
